# Estrogens and Progestogens in Environmental Waters: Analytical Chemistry and Biosensing Perspectives on Methods, Challenges, and Trends

**DOI:** 10.1021/acs.analchem.4c06796

**Published:** 2025-04-21

**Authors:** Anna Lalik, Julia Szreder, Mirosława Grymel, Sebastian Żabczyński, Sylwia Bajkacz, Mateusz Pielok, Mirosław Cieślik, Agnieszka Kicińska, Agata Wawrzkiewicz-Jałowiecka

**Affiliations:** † Department of Systems Biology and Engineering, Silesian University of Technology, Akademicka 16, 44-100 Gliwice, Poland; ‡ Department of Organic Chemistry, Bioorganic Chemistry and Biotechnology, Silesian University of Technology, B. Krzywoustego 4, 44-100 Gliwice, Poland; ¶ Department of Environmental Biotechnology, Silesian University of Technology, Akademicka 2, 44-100 Gliwice, Poland; § Department of Inorganic, Analytical Chemistry, and Electrochemistry, Silesian University of Technology, Krzywoustego 6B, 44-100 Gliwice, Poland; ∥ Faculty of Chemistry, Silesian University of Technology, Strzody 9, 44-100 Gliwice, Poland; ⊥ Department of Physical Chemistry and Technology of Polymers, Silesian University of Technology, Strzody 9, 44-100, Gliwice, Poland; # Biotechnology Center, Silesian University of Technology, B. Krzywoustego 8, 44-100 Gliwice, Poland

## Introduction

Estrogens and progestogens of natural and synthetic origin constitute a considerable group of endocrine-disrupting chemicals (EDCs) due to their persistence in the environment and the possibility of disrupting endocrine systems in wildlife and humans. It has prompted international regulatory institutions to introduce legislation mandating hormone determination in environmental samples and assessing their biological effects. Despite increased scientific evidence linking these chemicals with altered human health and their documented impact on fauna, many biological aspects of the long-term exposure and intake of sex hormones are still not completely known. Addressing these problems triggers dedicated research, which requires reliable analytical methods to quantify hormone concentrations in complex environmental matrices and assess their endocrine activity. However, the accurate detection and quantification of estrogens and progestogens pose considerable challenges, including sample stability during collection and transport, potential interferences, and trace levels of target analytes. Moreover, analytical protocols must be carefully tailored to specific research objectives and experimental designs. Therefore, this review provides a critical overview of the methods used for estrogen and progestogen analytics in liquid environmental samples. We focus on sample preparation, analyte detection, and trends in analytical chemistry and biosensing. Particular attention is paid to presenting contemporary strategies for environmental hormone analysis with their strengths and limitations, offering a practical guide for researchers, summarizing current practices, and highlighting emerging challenges in the field.

### Sex Hormones as Endocrine-Disrupting Chemicals

Environmental pollutants, known for their potential to significantly disrupt the endocrine systems of both humans and wildlife, pose a serious threat to health and overall quality of life. These substances are referred to as endocrine-disrupting chemicals (EDCs). The EDCs encompass a diverse range of chemicals from various sources such as bisphenols (plastics), phthalates (plasticizers), compounds found in pesticides, and naturally occurring endocrine hormones or their synthetic analogs.^1^ Exogenous sex steroid hormones, including estrogens and progestogens (also called progestins, progestagens, gestagens) being the main focus of this work, can act as potent EDCs in eukaryotes.^2−4^

Water is a major vector for EDC exposure in living organisms.^5^ Numerous scientific papers report that natural and synthetic sex hormones are present in aqueous environmental matrices all over the world with significant geographical variations (Table 1). For instance, in EU member states, the highest concentration of the following estrogens were detected in Doubs river (France) (29.2 ng/L, estrone (E1)), in Berounka river (Czech Republic) (4.6 ng/L, 17α-estradiol (αE2)), in Danube River (449 ng/L, 17β-estradiol (E2)).^6^ In turn, in Asia 17α-ethinylestradiol (EE2) and estriol (E3) were detected among others in Yellow River Delta and seawater (southern Bohai Sea) with concentrations in the range from 11.0 to 268 ng/L and 1.98 to 99.7 ng/L, respectively.^7^ These amounts are significantly lower in comparison to the levels of estrogens found in African countries. Concentration and range of EE2 (167 and 1739 ng/L), E2 (up to 5338 ng/L), and E1 (677 and 4152 ng/L) were recorded in the Ghanaian aquatic environments.^8^ These disparities^6−9^ underscore the challenges of conducting large-scale, harmonized studies on sex hormone pollution sources, which require reliable cross-country data. However, creating large-scale, relevant studies regarding the problem of the main sources of sex hormones in the environment is difficult because it requires reliable data from across multiple countries. Despite global scale research that identified livestock as the primary source of sex hormone pollution globally,^10^ human excretion is a significant contributor in regions like Europe, where livestock populations are declining.^6^ Hormones finding their way into surface waters are one thing, but getting rid of them is another. As long as wastewater treatment plants (WWTPs) are not capable of eliminating them to a satisfactory level, hormones will remain a significant issue. Even with a removal efficiency of 70–80%^11^ WWTPs dealing with huge amounts of water are bound to leave out harmful amounts of hormones leading to their accumulation.^12^

**Table 1:** Occurrence of estrogens and progestogens in environmental aqueous matrices of various countries according to selected reports (2014-2024)

Matrix (Country)	Methods of sample pretraetment and analysis	Detected values	Reference
Mineral water, runoff and wastewater	DLLME, MEKC-MS	<LOD	(13)
Drinking water, wastewater	SPE, HPLC-DAD	16–200 ng/L (E2 and EE2)	(14)
Wastewater (influent and effluent)	SPE, GC × GC–MS	<LOD (E2 and EE2)	(15)
Wastewater, groundwater, drinking water, river water	FPSE, HPLC-FD	1–10 ng/mL (spiked E2 and EE2)	(16)
River water (Turkey)	SPME, LC-MS/MS	0.10–7.53 ng/L (estrogens)	(17)
Underground, estuary, sea and wastewater (Portugal)	BAmM, LC-DAD	300–4300 ng/L (estrogens and progestogens)	(18)
Freshwater, lakes and rivers (Lake Balaton)	SPE, HPLC-MS/MS	0.23–13.67 ng/L (P); 0.85–3.40 ng/L (LNG); 0.26–4.30 ng/L (DRO)	(19)
Sea and fresh tap water (Belgium)	UHPLC-HRMS	0.26–39.0 ng/L (estrogens, progestogens)	(9)
River water (France, Belgium)	SPE, GC-MS	34.2–116.2 ng/L (E1); < LOQ-3.6 ng/L (E2); < LOQ-77.7 ng/L (E3); < LOQ (EE2); < LOQ-10.9 ng/L (P)	(20)
River and wastewater (Switzerland)	SPE, LC-ESI-MS/MS	2.3–37 ng/L (E1); n.d.–8.7 ng/L (E2); 2.0–22 ng/L (P); n.d.–66 ng/L (17OHP)	(21)
Tap, surface and wastewater (Canada)	SPE, UHPLC-MS/MS	0.80–790 ng/L (estrogens, progestogens)	(22)
Raw and treated wastewater (Poland)	SPE - triple-sorbents, GC/MS	<MDL (E1); < MQL (E2); < MQL (T); < MQL (EE2); < MDL (E3)	(23)
Surface and wastewater (Argentina)	SPE, HPLC-MS/MS	1.54–85.15 ng/L (E1); 0.94–7.06 ng/L (E2); 0–64.24 ng/L (EE2); 0.40–384–43 ng/L (E3); 0.32–6.85 ng/L (17OHP); 2.55–12.73 ng/L (P)	(24)
Surface water (river, canal USA)	online SPE-LC-HRMS, APCI	8.9–29.8 ng/L (E1); 16.8–34.0 ng/L (E2); 3.6–5.8 ng/L (EE2)	(25)
Livestock farms WWTPs	SPE, LC-MS/MS	338.24 ng/L (EE2), n.d.-12.7 ng/L (PG)	(26)
Surface and wastewater tap, mineral, well, pond, swimming pool, river, waste (Spain)	SPE, GC-MS	14.0–110 ng/L (E1); 3.3–22.0 nl/L (E2); 4.0–12.0 ng/L (EE2); 13.0–24.0 ng/L (P)	(27)
Drinking water, mineral water (Hungary)	SPE, HPLC-MS/MS	<MDL (TST, E1, E2, E3); 12–109 pg/L (EE2)	(28)
Surface water (Brazil)	ATPSs based on MeCN + double PIL, UHPLC	9.3–16.2 ng/L (E2); 11.2–14.6 ng/L (E3); 13.9–29.2 ng/L (EE2); 10.6–11.2 ng/L (P)	(29)
Drinking water (Italy)	TF-ME, HPLC-MS/MS	8.7 ng/L (E1); 14.1 ng/L (E2); 4.5 ng/L (P)	(30)
Wastewaters (Italy)	TF-ME, HPLC-MS/MS	17.4 ng/L (E1); 25.4 ng/L (E2); 8.2 ng/L (P)	(31)

### Regulatory Framework for Monitoring Estrogenic Micropollutants in Aquatic Environments

Water pollution by sex hormones is a global problem. It has found its imperfect reflectance in water quality policies, which in many regions worldwide have significant gaps in addressing hormone-related environmental risks. The regulatory frameworks for mitigating water pollution are evolving unevenly and differ from country to country. Nonetheless, they sometimes demonstrate a science-policy feedback loop because the growing scientific evidence highlighting sex hormone pollutants as environmental threats can progressively shape legislation acts.

As a running example, we briefly consider the EU regulations concerning the monitoring of micropollutants, particularly estrogens, in aquatic environments. One of the first European projects called “Assessment of technologies for the removal of pharmaceuticals and personal care products in sewage and drinking water facilities to improve the indirect potable water reuse (POSEIDON)”^32^ funded under Programme for research, technological development, and demonstration on “Energy, environment and sustainable development, 1998–2002” and launched in 2001 included EE2 assessment. Since then, many scientific articles have been published, revealing that substances from the estrogen group pose significant environmental challenge. Therefore, the European Union has implemented guidelines and strategies to manage the issues of various micropollutant groups in aquatic systems. Nonetheless, Directive 2008/105/EC of the European Parliament and of the Council of 16 December 2008^33^ and amended by Directive 2013/39/EU of the European Parliament and of the Council of 12 August 2013^34^ on environmental quality standards in the field of water policy did not include estrogens. In 2015, a watch list of substances for Union-wide monitoring in the field of water policy under Directive 2008/105/EC of the European Parliament and of the Council was established by the European Commission.^35^ This list included substances for which available information suggested that they may pose a significant risk to the aquatic environment at Union area but where monitoring data was insufficient to determine their actual risk. It includes estrogens (EE2, E2, E1) and additionally indicates the types of metrics for monitoring and possible methods of analysis which do not create excessive costs. EE2, E2, and E1 were still present on the watch list amended in 2018.^36^ However, they vanished in 2020 and 2022 indicating a gap in consistent oversight. It was related to the fact that the duration of continuous monitoring for each substance on the watch list shall not exceed four years. The new proposal for a Directive of the European Parliament and of the Council amending Directive 2000/60/EC establishing a framework for Community action in the field of water policy, Directive 2006/118/EC on the protection of groundwater against pollution and deterioration and Directive 2008/105/EC on environmental quality standards in the field of water policy^37^ contains a modified list of priority substances called ‘Environmental quality standards (EQS) for priority substances in surface waters’ which includes assessment of E1, E2, and EE2 as an annual average value (AA EQS) and as a maximum allowable concentration (MAC EQS). Where the MAC EQS values are marked as “not applicable”, the AA EQS values are considered protective against short-term pollution peaks in continuous discharges, since they are significantly lower than the values derived based on acute toxicity. Unless otherwise specified, AA-EQS and MAC EQS apply to the total concentration of all substances and isomers in inland surface waters, which encompass rivers and lakes and related artificial or heavily modified water bodies. Additionally, the proposal obliges Member States to apply effect-based methods (EBMs) to assess the cumulative effects of hormones on organisms. EBMs, which include bioanalytical methods using the response of whole organisms (*in vivo*) or cellular bioassays (*in vitro*) to detect and quantify the effects of groups of chemicals on toxicological end points of concern, can serve as a useful complement to chemical monitoring by providing an integrated measure of the ’chemical health’ of the aquatic environment.

The mentioned example of EU regulations underscores the challenges of long-term pollutant monitoring, policy continuity, and the impact of science on policy. The last one, at the collective level, may have tremendous consequences due to the possible inclusion of particular EDC substances in legislation documents, the adoption of certain methodologies there, and the commissioning of specific monitoring modes, e.g., by interactive and cumulative parameters. From the perspective of research in academia, establishing sound and unbiased procedures for cleanup, preconcentration, and determination of analyte (e.g., hormone) is mandatory for relevant and reliable data under investigation. At the same time, the development of such procedures can shape, to some extent, the legal regulations on monitoring concentration levels of pollutants in the environment.

### Health Risks to Wildlife and Humans from Exposure to Estrogen and Progestagen Traces

It is worth briefly considering the sex-hormone-related health risks in wildlife and the related environmental challenges mentioned before. Many laboratory ecotoxicological experiments conducted with various organisms (e.g., fish, amphibians) have demonstrated that certain steroid hormones, both natural and synthetic, can negatively impact reproduction even at ng/L levels as detected in surface waters.^38^ Even low concentrations of EE2 cause a range of undesirable effects, leading to reduced egg production by females and feminization of males. For example, EE2 and synthetic progestins significantly increased ovipositor length, reduced the prominence of secondary sexual characteristics such as tubercle number and prominence, and reduced plasma 11-ketotestosterone concentrations in tests using fathead minnow (*Pimephales promelas*) at low environmentally relevant concentrations (nanograms per liter).^39^ Brion et al. exposed zebrafish (Danio rerio) for 3 weeks to low concentrations of E2 including environmentally relevant concentrations (5, 25, and 100 ng/L).^40^ Exposure of adult fish to E2 led to alterations in secondary sexual characteristics in males at concentrations of 25 and 100 ng/L, along with a dose-dependent suppression of egg production. Generally, this study indicated that the intensity of E2 on the zebrafish reproductive effects depends on the duration and concentration of exposure. Some of these effects, such as those on sexual differentiation, are permanent, while others, such as the impact on Vitellogenin induction, are reversible.

The continuous exposure to EDC, including sex hormones, is an increasing global problem in the environmental context (effects on fauna) and also from the perspective of human health hazards.^41,42^ Excess intake of sex steroids deranges their normal physiological effects by affecting hormonal homeostasis (synthesis and/or metabolism), altering hormone receptor levels and their signaling pathways, which can lead to systematic effects like developmental alterations, changes of functional and behavioral sexual characteristics, metabolic disorders,^43^ and perturbation of mood and stress control,^44^ immunity and disease susceptibility,^45−49^ and cancer risk.^50−52^

### The Need for Reliable and Well-Established Procedures in Estrogens and Progestagens Analytics

Both in the scientific community and in public awareness, knowledge about environmental pollution by sex steroids and its consequences including the sex-hormone-related health hazards in wildlife and humans is growing. Still, however, a comprehensive understanding of the long-term effects and molecular mechanisms of hormone disruption by exogenous estrogens and progestogens is not fully unraveled. Additionally, understanding the behavior, persistence, and impact of these hormones in water systems is crucial for developing effective regulations for environmental protection and mitigation of the related risks. This stimulates dedicated versatile multidisciplinary research, which requires reliable, well-established procedures for sample collection, transportation, and sex hormone determination fulfilling the needs of a planned experiment. The development of such methodologies and effective strategies for their optimal case-specific choice can be considered a challenge for analytical chemists, biochemists, and molecular biologists.

This review aims to gather and present in accessible form the most relevant contemporary techniques and methods of estrogen and progestogen analytics in environmental aqueous matrices to accelerate research for all parties taking up the topic. The choice of analytical methods for both sample preparation, including analytes extraction, preconcentration, and cleanup, as well as hormone levels’ determination can be quite challenging in laboratory practice. We provide a detailed overview of the experimental techniques used for sample preparation and estrogen and progestagen determination using modern techniques of instrumental analytical chemistry, immunoassays, or biosensors. Particular attention is paid to the pros and cons of each method. We also discuss the question of potential interferences, the complications related to the trace levels of analytes, and their stability considering the effects of temperature, pH, and photo- and biodegradation. The choice of a particular sample preparation and analyte detection method often depends on the required sensitivity, the complexity of the sample matrix, the available instrumentation, and the research team’s expertise. We also briefly describe the second important perspective, the question of reliable biological potency evaluation by bioassays. The determination of overall sex hormone load via functional assays gives complementary information to quantification of hormone concentrations, while monitoring environmental samples. This review provides a valuable critical summary, which may foster well-tailored sample preparation and analyte determination strategies. This work also outlines the recent trends and challenges in the context of estrogen and progestagen analytics of environmental water samples.

## Difficulties in Reliable Quantifying Hormone Concentrations in Environmental Waters

Accurate quantification of hormones in environmental waters is challenging due to their low concentrations and complex matrices. Below we discuss the essential aspects of proper sample preparation and reliable hormone determination.

### Trace Levels

Hormones in environmental waters are most commonly found in the nanograms per liter (ng/l) range (Table 1), requiring highly sensitive analytical methods. Hence, tackling the challenge of reaching high selectivity and sensitivity in measurements is essential for environmental analysis.^53^ Standard analytical techniques may not have sufficient sensitivity to detect these low concentrations reliably. Even highly sensitive instruments have detection limits, making hormone quantification below these thresholds uncertain. Larger sample volumes are often needed to collect trace amounts of hormones. However, handling large volumes poses challenges including an increased risk of sample loss and contamination. At such low concentrations, even minor contamination from laboratory reagents or external sources can distort the results.

### Complex Matrices

Occurrence of pollutants (e.g., estrogens and progestagens) in the environment creates a necessity of quantifying them in complex matrices which contain widespread impurities, microorganisms, and interferents. For this reason, generally, the first pivotal step of sample pretreatment is filtration. For example, it prevents sorbents from being blocked by suspended solids during solid-phase extraction (SPE). Thus, filtration is a crucial step in sample preparation. It is important to note that estrogens, particularly in wastewater analysis, tend to adsorb onto particulate matter due to their high *K*_*d*_ value. The sorption coefficient *K*_*d*_ describes the solid–liquid partitioning characteristics of a compound. Studies have shown that the sorption of micropollutants in WWTP is generally insignificant (<10%) with *K*_*d*_ below 0.3 L/gSS. For example, the *K*_*d*_ value determined for EE2 closely approximates this threshold, measuring 0.28 ± 0.005 L/gSS for primary sludge and 0.35 ± 0.04 L/gSS for secondary sludge.^54^

Troublesome interferences, which can influence hormone stability, are reactive oxygen species (ROS), triplet state dissolved organic matter (^3^DOM*), and pH of the sample. Other matrix effects may vary depending on analytical technique. In immunoassays, cross-reactivity of antibodies can entail errors in analysis.^55^ In chromatographic quantification, main interferents are other steroid hormones and lipophilic substances.^56^ The most widely used countermeasure is to separate the analyte from the matrix as effectively as possible. This also prevents potential decomposition, especially the microbial one. For this purpose, solid phase extraction is mainly used.^57−59^ However, techniques such as liquid–liquid extraction (LLE),^60^ immunoaffinity extraction,^61^ or quick, easy, cheap, effective, rugged, and safe (QuEChERS) approach^62^ are also utilized. All of these analyte separation and preconcentration methods are discussed in more detail later in this review.

### Stability

Hormones in aqueous solutions, particularly in the presence of microorganisms, can degrade through various pathways. Although the primary focus of this work is on methods for quantifying hormones, it is also essential to develop strategies to prevent their decomposition and minimize analyte loss. EDCs, including sex hormones, found in environmental waters and wastewater can react with other chemicals present in these matrices and form new derivative products.^63^ In turn, the concentration, variability, and behavior of hormones also vary depending on the season of the year^64^ as the environmental water characteristics (e.g., contaminations, physicochemical parameters) and the microbial activity.^65,66^ Properties such as stability in different environments and the formation of degradation products require further clarification.

#### Photodegradation

Hormones exposed to sunlight can undergo photodegradation in two ways: directly and indirectly. Direct photodecomposition occurs when chemical reaction is triggered by photon absorption of the hormone molecule, while indirect decomposition is caused by ROS or triplet dissolved organic carbon (3DOC*).^67^ ROS and 3DOC* can be formed as a result of light interaction with organic matter dissolved in water.^68^ Subsequently, hormone molecules are attacked by ROS or 3DOC*, usually leading to their oxidation.^67^ While both ways of photodegradation are possible in the environment, kinetic studies show that photodegradation in the presence of dissolved organic matter is significantly faster.^69−71^ Due to possible loss of quantified hormones, samples should not be exposed to sunlight prior to analysis.

#### Thermal Degradation

In the study,^72^ solutions of 17β-estradiol were stored at room temperature, refrigerated (4 °C), and frozen (−20 °C) for 24 and 48 h. The high-performance liquid chromatography coupled with ultraviolet detection (HPLC-UV) hormone determination was carried out to evaluate hormone stability in the aforementioned conditions. The results suggest that hormones themselves are stable and do not undergo destruction under a temperature of ∼25 °C. However, hormones found in surface waters are indirectly affected by temperature. E1, αE2, and E2 in a lagoon water matrix were tested under 35 °C over several time periods.^73^ Amounts of hormones remaining in solution after 52 days ranged around 77–85%, suggesting their relative stability. The dominant effect leading to degradation was temperature-dependent bioactivity in water samples.^73^ In environmental water samples, not only pure hormones but also hormone conjugates such as ethinyloestradiol 3-sulfate or 3-glucuronide are present. Testing several different conjugated steroid estrogens proved that a decrease in temperature leads to their slower degradation in sewage water.^74^ There is a direct correlation between the temperature and the deconjugation process. De Barros et al. studied the stability of estrogens (αE2, E2, E3, E1, EE2, and diethylstilbestrol (DES*)), progestogens (progesterone (P), norgestrel (NG), norethisterone (NTR), and megestrol acetate (MGA)), androgens (androstenedione (A) and testosterone (TST)), and prostaglandins (cloprostenol).^75^ For each compound, the stability of the standard solution (10 μg/L) was assessed under different storage conditions: (i) at −18 °C for up to 60 days, (ii) at 4 °C for up to 15 days, and (iii) at room temperature (20 °C) with or without exposure to light for up to 7 days. These compounds were stable for 60 days at −18 °C, 7 days at 4 or 20 °C.^75^

#### Microorganisms

One of the main factors responsible for hormone decomposition in the environment is the influence of microorganisms.^76^ This is a significant problem during the quantification of hormones in environmental waters, where a matrix rich in bacteria causes high analyte losses. There are plenty of bacteria species that can contribute to analyte degradation.^77^ Many of them occur in waters, causing both direct and indirect degradation of hormones, through the production of dissolved organic matter.^67,78^ Due to the natural occurrence of many different microorganism species in environmental waters, ensuring analyte stability tends to be difficult. Therefore, it is advised to quantify hormones as quickly as possible after sample collection.

#### Influence of pH

Considering the impact of the environment on hormone molecules, pH seems to impact mostly their solubility and does not directly lead to their degradation. The three tested hormones E1, E2, and EE2 tended to be more soluble at pH above 7.^79^ Hormones having ionizable functional groups are sensitive to pH levels, which may impact the ability to quantify them in methods requiring the polarity of a molecule. The entire environment of hormones in a system is influenced by its pH, which may also lead to additional adsorption effects on other bulk organic matter, which leads to failure in isolating them from wastewater. It was observed that absorption to molecules like humic acid or tannic acid can vary depending on pH levels for estrone and progesterone due to carbonyl groups being strong hydrogen acceptors.^80^

Determining hormones in environmental waters is a complex task. From the moment of sampling, proper preparation is essential to ensure reliable results. This process must be efficient while maintaining appropriate storage conditions such as low temperatures and darkness.

## Sample Preparation

After the environmental sample is collected, the next step is to properly prepare and concentrate it. Most of the hormones are present in environmental water samples at trace levels. Thus, determination of their content requires appropriate preparation of the sample to minimize the influence of the matrix and increase analyte concentration. The first step involves filtration due to the high levels of suspended solids and organic matter in environmental waters. Aqueous matrices can be efficiently filtered through different materials, among which the most popular are glass fibers. Other materials commonly used are nylon, cellulose nitrate, or polymer filters, e.g., from poly(vinylidene fluoride).^81,82^ Besides filtration, the analytical sample preparation should embrace extraction of the analytes, preconcentration, and cleanup. Sample derivatization can be also needed (e.g., for hormone determination by gas chromatography coupled with mass spectrometry (GC-MS)).

Sample preparation before analysis is a crucial step and is often the primary source of errors in quantitative analysis. In the further part of this review, we describe versatile analyte preconcentration methods that are compatible with certain quantification techniques.

### Analyte Preconcentration

Estrogens and progestogens are typically found in real water samples at trace or ultratrace levels.^83,84^ Most available analytical methods lack sufficient sensitivity, making preconcentration an essential step in the quantitative analysis of sex hormones. The choice of sample preconcentration and purification techniques depends on the properties and concentration of the analytes, the complexity of the matrix, and the final analytical method used for hormone quantification. Below we highlight the commonly used robust preconcentration methods together with appropriate examples of their practical use.

#### Solid-Phase Extraction

Considering the sex hormone enrichment efficiency, the classical SPE hydrophobic sorbents (C_8_, C_18_) retain analytes by nonselective hydrophobic interaction, which, however, leads to coextraction of high amounts of matrix interferences.^85^ To minimize this drawback, over the years, more advanced SPE technologies have been developed. Among the large variety of available advanced sorbents on the market, particular attention could be paid to hydrophilic–lipophilic balance (HLB) sorbents, e.g., Oasis HLB (copolymer of *N*-vinylpyrrolidone and divinylbenzene). The HLB sorbents are the most commonly used sorbents in SPE of aqueous environmental samples for natural and synthetic hormones determination,^21,27,86,87^ and it is recommended in US EPA method 539.1 for determination of hormones in drinking water.^22^ The HLB sorbents can be used for simultaneous extraction of different hormones in their mixtures over the entire range of pH,^21,27,86,87^ which, depending on the context of the hormone determination, can be considered as its advantage or drawback, because the greatest limitation of the HLB sorbents is their nonselectivity.^85^ It burdens the sex hormone analytics by the matrix effects due to the coextraction of the matrix components together with the analyte(s). To cope with this problem, through the years, new customized SPE solutions for estrogen and progestogen extraction have been developed, including methodological advancements, like the application of solid-phase microextraction (SPME). SPME can be considered as a miniaturized solvent-free variant of SPE, where frequently analytes can be adsorbed onto the inner coating/filling of a capillary tube (in-tube SPME; e.g.^88^) that is then directly introduced into the analytical chromatographic system. Significant progress in the development of effective sex hormone preconcentration techniques has been also achieved by introducing novel supporting formats and advanced sorbents, including functionalized metal–organic frameworks (e.g., MIL-101(Cr)-NH_2_,^89^ NH_2_-MIL-101(Al)^90^), magnetic nanoparticle-based materials (e.g., ethylenediamine-functionalized magnetic carbon nanotubes (EDA@Mag-CNTs),^91^ graphene oxide-maghemite (GO/γ-Fe_2_O_3_) nanoparticles,^92^ reduced graphene oxide/ZnFe_2_O_4_composite,^93^ β-cyclodextrin magnetic activated carbon composite,^94^ magnetic polyimide nanosheet microspheres,^95^ rubber-Fe_3_O_4_@SiO_2_@Mg–Al layered double hydroxide^96^), macroporous neutral polymer sorbents (e.g., IonPac NG1^97^), molecularly imprinted polymers (MIPs) (e.g., P-imprinted polymerized acrylic acid,^98^ E2-imprinted MAA-based polymer, and E2-imprinted 3-(trimethoxysilyl)propyl methacrylate,^99^ ready-to-use MIP-based cartridges Affinimip SPE Estrogens from Affinisep (Val-de-Reuil, France)^100−103^), and functionalized mesoporous silicas (e.g., mesoporous silica functionalized with octadecyl groups (SBA-15-C18)^101,104,105^) to list just a few of the possible approaches and the corresponding practical examples. The usage of multiple-sorbent solid-phase extraction can also efficiently improve the performance and reliability of hormone quantification in real-life samples.^23^ Additionally, there are also interesting novel methodological approaches to SPE, like electrochemically controlled SPE. As an example of that approach, the sorbent in the form of conductive polypyrrole coating of a steel mesh surface was obtained.^106^ From the two resulting morphologies, i.e., globular and nanotubular, the second turned out to be effective for extraction of progesterone in river samples, allowing to reach LOD = 16.7 μg/L and linear range from 50 to 500 μg/L when coupled with GC-MS. Another promising direction in the SPE of sex hormones in environmental water samples is to use membranes modified with conductive polymers and their derivatives. For example, polypyrrole was modified on the surface of cellulose membrane, which was adopted as a functional layer.^107^ When the optimized composite membrane was used with GC-MS for the determination of 14 contaminants in river water including E1, E2, E3, EE2, and P, it enabled to achieve satisfactory recoveries (107.8 ± 9.6% for E2, 108.6 ± 6.8% for E1, 102.8 ± 12.7% for E3, 107.1 ± 12.1% for EE2, 107.9 ± 21.8% for P).^107^

Considering the interesting strategies in the solid phase extraction of sex hormone samples, two types of adsorbents deserve particular attention and broader consideration. They are MIPs and magnetic/magnetically modified adsorbents (and the joint approach). Because the MIPs bear specific recognition sites for target molecules, they become reasonable, tailored alternatives to conventional SPE sorbents for the selective extraction and preconcentration of hormones in complex matrices.^108−111^

The first reports mentioning MIP-SPE in estrogens’ analytics are dated 1998,^112,113^ and they already indicated high selectivity of MIPs for the considered sex hormones. For example, MIP obtained by thermal polymerization of 4-vinyl pyridine (monomer) in the presence of E2 (template), ethylene methacrylate (cross-linker), and 2,2’-azobis(2,4-dimethylvaleronitrile) (initiator) showed high affinity and selectivity for E2 (retention factor ∼ 12 for E2).^112^ Nevertheless, the obtained material exhibited only moderate extraction efficiency for other estrogens, including 17α-estradiol, estriol, and estrone. Another MIP, synthesized using methacrylic acid as the functional monomer, ethylene glycol dimethacrylate as the cross-linking agent, E2 as the template, and 2,2’-azobis(isobutyronitrile) as the initiator, demonstrated satisfactory extraction efficiency for E2 (retention factor ∼ 5).^113^ However, this sorbent did not enable complete separation of sex hormones when their mixture was analyzed—particularly in the case of an equimolar E2 and TST mixture.

The later studies on MIP-SPE in sex hormone analytics showed considerable methodological improvements in selected aspects of analyte extraction. For example, a polymer synthesized from methacrylic acid (MAA) as the functional monomer, ethylene glycol dimethacrylate (EGDMA) as the cross-linker, and EE2 as the template, in acetonitrile as polymerization solvent, turned out to be effective in selective extraction of ethynylestradiol from river water samples.^114^ It enabled reaching as high EE2 recovery as 75% with RSD value of 8.6%. The possible interferences by other estrogens (E1, E2, and E3) can be efficiently excluded during the washing of the MIP-SPE column by toluene.

The MIP-based sorbents turned out to be quite effective for the selective extraction of progesterone from water samples. The performance of the progesterone-imprinted sorbent was optimized by testing different combinations of radical polymerization parameters, including the monomer (acrylic acid), solvent (acetonitrile), cross-linker (1,4-divinylbenzene), and template (P) ratio.^98^ The retention capacity of the obtained MIPs was determined using HPLC and ranged from 32.5% to 96% for progesterone.

MIPs were also used as sorbents for extracting 17β-estradiol, estriol, and estrone from water samples.^99^ In one method, MIP sorbents were synthesized via bulk polymerization using methacrylic acid (MAA) as the functional monomer, divinylbenzene as cross-linkers, and a mixture of acetonitrile and toluene (3:1, v/v) as the porogen. Coupled with HPLC and a DAD detector, the recoveries achieved were 74.3 ± 0.9% for E1, 82.2 ± 0.3% for E2, and 82.0 ± 0.5% for E3, respectively. In the same study, surface imprinting was conducted to produce 3-(trimethoxysilyl)propyl methacrylate (γ-MAPS) film on porous silica gel, yielding recoveries comparable to those of bulk-polymerized MIP for E1 and E3, with R_γ–*MAPS*_*E*_2_ = 72.0 ± 1.3%. However, the authors highlighted the advantages of the γ-MAPS-silica gel sorbent, specifically eliminating the fractionation stage, thereby reducing the column preparation time and minimizing fractionation losses. Better extraction efficiency was reported for the MIP based on 3-(trimethoxysilyl)propyl methacrylate and adhered to porous silica gel of pore size 60 Å. It enabled over 90% recovery of hormones (estrone, 17β-estradiol, estriol, 17α-ethinylestradiol, progesterone, and testosterone) in environmental water samples.^115^

A novel polymer was proposed for determining trace estrone in environmental samples using estrone as a template, 3-aminopropyltriethoxysilane as the functional monomer, and tetraethoxysilane as the cross-linker. Unlike most previous approaches, this method avoided acrylate-based polymers, which are complex and require inert conditions. The proposed material - a molecularly imprinted microsphere, synthesized by surface molecular imprinting technique combined with a sol–gel process, showed high selectivity for estrone, with a distribution coefficient approximately four times higher for estrone than for 17β-estradiol, estriol, and diethylstilbestrol. When MISPE was combined with HPLC, the recoveries of estrone from water samples ranged from 86% to 95% with an RSD of 9.8%.^116^

Of considerable interest for a number of studies is the application of commercially available MIP sorbents in SPE for sex hormone analytics. The ready-to-use MIP-based cartridges Affinimip SPE Estrogens from Affinisep (Val-de-Reuil, France) were applied to perform the solid phase extraction of three estrogens (estriol, 17β estradiol, and 17α-ethinylestradiol) and two metabolites of E2 (17β-estradiol-glucuronide and 17β-estradiol-3-methyl ether) in wastewater samples (collected from a veterinary hospital as well as the influents and effluents of a wastewater treatment plant in Gran Canaria, Spain).^100^ The MIP-SPE was coupled with UHPLC and FL detection to determine the analyte concentrations. Using an optimized MIP-SPE procedure (i.e., 3 mL of acetonitrile and 3 mL of ultrapure water at equilibration (2 drops/s), 50 mL of wastewater at pH = 9 at loading (0.5 drops/s), 3 mL of ultrapure water at washing of interferents (1 drop/s), application of vacuum or nitrogen flow through cartridge during 3–5 min drying, 1.5 mL of methanol at elution (1 drop/s)) yielded satisfactory recoveries and the determination results for the analytes in wastewater samples, with over 60% recovery and low RSDs (under 10%) for most compounds in all samples. The Affinimip SPE Estrogens cartridges have also been earlier utilized in the studies of Lucci et al.,^101^ where the estrogens (E1, E2, αE2, E3, EE2, dienestrol, and diethylstilbestrol) were determined in Milli-Q, river, and tap water samples. Here, however, different procedures were applied. Before each use, sorbents were conditioned with acetonitrile (5 mL), followed by water (5 mL). In turn, during the MIP-SPE experiments, the samples were passed through the MIP-SPE cartridge at a flow rate of 2 mL/min. The sorbent was washed with 4 mL of water/acetonitrile (80:20, v/v) followed by 2 mL of water. Drying of the polymer was performed by using a full vacuum for 5 min. Then, the sorbent was washed with acetonitrile (2 mL) followed by 2 mL of an acetonitrile/methanol mixture (95:5,v/v). Estrogenic compounds were finally eluted from the cartridges with methanol (3 × 1 mL). When the MIP-SPE was coupled to UPLC-MS/MS for estrogen determination, recoveries over 82% have been achieved for E1, E2, αE2, E3, EE2 for all sample types. Notably, the proposed MIP-SPE showed higher recovery for most hormones compared to C_18_ SPE, except for dienestrol and diethylstilbestrol, where C_18_ SPE was more efficient.^101^ The same MIP sorbent (Affinimip SPE Estrogens, Affinisep, Val-de-Reuil, France) was used in the online MIP-SPE coupled to an LC-MS/MS system proposed for the determination of hormones in water and sediment samples.^102^ Before application, the sorbent was passed through a 25 μm sieve and then was packed into a PEEK 30 × 2.1 mm column (VICI Jour, Schenkon, Switzerland) using a slurry packer kit (VICI Jour). The executed procedure included: 3 min of sample loading at a rate of 0.125 mL/min, 2.5 min washing with acetonitrile at a flow rate of 0.35 mL/min, cleaning with methanol for 9.5 min at 0.35 mL/min, and 5 min conditioning with ACN at 0.35 mL/min. Coupled with LC-MS/MS, this method yielded recoveries of 92–105% and precision of 4–6% for E1, E2, E3, EE2 in water and sediments samples from the Svratka River (Brno, Czech Republic).^102^ The authors noted that the most important parameter controlling the analytes’ retention was the sample flow rate. The increase of sample flow rate from 0.125 to 0.25 mL/min resulted in the loss of E1 and EE2 up to 30%, while at flow rate of 0.5 mL/min and higher, none of the analytes was quantitatively retained. In the study of González and Cerdà,^103^ another online SPE system based on the Affinimip SPE Estrogens sorbent has been proposed. This sequential injection analysis-lab on valve system (SIA-LOV-HPLC-DAD) used only 15 mg of MIP resin (compared to 100 mg in previous studies^100−102^)) for the selective microsolid phase extraction (μSPE) of the estrogens (E1, E2, E3, EE2) in wastewaters in a fully automated way. The μSPE procedure parameters were as follows: loading 5.0 mL of sample at 0.5 mL/min, washing by 0.5 mL of water at 1.0 mL/min, elution by 0.4 mL of MeOH at 1.0 mL/min, and reconditioning by sequentially aspirating and passing through the column 1.0 mL of ACN and 1.0 mL of water at 1 mL/min. The MIP-μSPE method achieved high efficiency, with recoveries of 94–113% for E3, 94–108% for E2, 88–112% for EE2, and 81–105% for E1.^103^

Significant methodological progress considering the solid phase extraction has been made by the introduction of the magnetic solid phase extraction (MSPE).^117−120^ Among them, the magnetic surface molecularly imprinted polymers (MMIPs) are prominent representatives.^119,120^ In recent years, a plethora of studies has been conducted to develop advanced MSPE materials. Here, we focus on a selection of studies that highlight promising directions in this field.

Considering favorable core materials, mesoporous magnetic materials are good representatives due to their complex structure and morphology ensuring appropriate cavities in nanothick matrix wall for polymer-shell imprinting, many accessible binding sites for the target molecule, which eventually leads to good analyte recognition by and fast mass transfer. In this regard, magnetic mesoporous silica particles Fe_3_O_4_@SiO_2_@mSiO_2_ coated by selected molecularly imprinted polymers allowed to achieve highly sensitive and selective preconcentration of estrogens from water samples.^121,122^ Other materials with large surface areas and inherent porous structures have proven to be suitable for synthesizing magnetic sorbents. For instance, the combination of activated carbon and cyclodextrin was used to produce a magnetic composite, successfully applied as an adsorbent for extracting and enriching E1, E2, hydrocortisone, and progesterone from wastewater and river water samples, achieving high recoveries of over 97.5 ± 3.1.^94^ Similarly, good dispersibility and large specific surface area underlaid the usability of magnetic polyimide nanosheet microspheres in solid-phase extraction for trace analysis of estrogens in aqueous samples by GC-MS.^95^ A different high-performance sorbent was recently proposed by Matin et al.^96^ The rubber-Fe_3_O_4_@SiO_2_@Mg–Al layered double hydroxide (LDH), joins the benefits of the distinct structural LDH framework, which gives a complex two-dimensional brucite-like layered molecular architecture enabling for the capture of steroid hormone molecules in its interlayer spaces and ferrimagnetic properties of the core. This material enables enrichment of trace amounts (LOD in the μg/L regime) of E1, E2, and bisphenol A (BPA) from aqueous samples. Application of another advanced magnetic material of the intricate internal structure was reported by Wang et al.,^123^ where lightweight daisy-like magnetic molecularly imprinted polymers were obtained via chemical etching and E2 template immobilization. This material exhibited strong magnetic responsiveness, light mass, and plentiful imprinted sites in the etched channels, resulting in high adsorption capacity for E2 and excellent selectivity. Application of this sorbent allowed us to reach a low LOD in E2 analysis of 28 ng/L after combining with HPLC and 94.4–97.8% E2 recovery.

Another approach involves designing hydrophilic magnetic molecularly imprinted nanobeads to target 17β-estradiol.^124^ The central idea was to apply E2 imprinted polydopamine layers on carboxyl-group-functionalized Fe_3_O_4_ nanoparticle carriers. The E2–dopamine ”imprints” guaranteed effective interactions of the MIP layer with E2 through hydrogen bonding and π–π stacking. Thus, the proposed material allowed for efficient enrichment and HPLC-UV detection of E2 in environmental water samples (LOD = 0.008 μg/L). Ensuring effective noncovalent interactions between analyte and sorbent, which favor the separation process by MSPE, was also a key concept of Ferreira et al.^92^ In their work, graphene oxide-maghemite (GO/γ-Fe_2_O_3_) nanoparticles, synthesized via coprecipitation, have been used for extraction of E2 and EE2 in tap water samples. The GO/γ-Fe_2_O_3_ composite has many functional groups, including hydroxyl (−OH) and epoxides (C–O–C) on the basal plane with carbonyl (>C=O) and carboxyl (−COOH) groups at the edges, which can effectively coordinate the steroid analytes. This sorbent allowed for a satisfactory recovery for both hormones ranging from 83 to 98% with ∼ 7% precision when determined with HPLC-FD.

Another example of E2 analytics in environmental water samples is the study of Hao et al.,^125^ where the water-compatibility of the magnetic molecularly imprinted nanoparticles used for selective extraction of E2 was a core idea. An interesting concept implemented in this work was to use a nontoxic, nonimmunogenic, and biodegradable biomacromolecule, gelatin, for three functions: interacting with a template to obtain a template-monomer complex, assembling covalently at the surface of aldehyde-modified magnetic nanoparticles to form imprinted shells, and improving the water-compatibility of materials for attaining high extraction efficiency in water matrices. The prepared nanomaterials exhibited high adsorption capacity and favorable selectivity and turned out to be a material that can be successfully applied in the specific isolation and determination of E2 from environmental water samples (after coupling with HPLC-UV, the LOD limit was 0.04 ng/mL, and recovery ranged from 88.3% to 99.1% with the RSDs less than 7.2%).

Magnetic nanocomposite adsorbents can be exploited by simultaneous determination of a considerable number of hormones in environmental aqueous samples.^91^ The application of ethylenediamine-functionalized magnetic carbon nanotubes (EDA@Mag-CNTs) as SPE sorbents and their coupling with the UFLC-MS/MS detection system allowed for fast and reliable determination of 24 steroid hormones (including, e.g., progesterone) in river water samples.

Another MSPE-based preconcentration method is described by Lekota et al.,^126^ where a ferric oxide-aluminum oxide carbon nanofiber nanocomposite (Fe_3_O_4_–Al_2_O_3_@CNFs) was used as a nanosorbent in ultrasound-assisted dispersive magnetic solid-phase extraction (UA-DMSPE) of E2 in wastewater samples. E2 quantification was achieved using HPLC-DAD, and the obtained recoveries ranged from 93.0 to 99.3%. The detection limit was 0.025 μg/L, and the limit of quantification was 0.083 μg/L.

The use of MSPE has also been shown to be an efficient technique for the extraction and enrichment of estrogens and progestogens in the case of more demanding matrices, as shown by Li et al.,^93^ who not only analyzed river waters but also soil and fish samples (the analysis of such matrices exceeds, however, the merit of this work).

#### Liquid–Liquid Extraction

Traditional liquid phase extraction usually requires large amounts of organic solvents, which can lead to significant analyte losses, prolongation of the analytical procedures, increasing their costs, and exhibiting negative environmental impacts.^127,128^ Despite that, LLE could sometimes be considered a reasonable alternative to SPE, especially in cases dealing with nonpolar analytes (e.g., lipophilic sex hormones). The overall performance of hormone separation from environmental samples depends on the distribution coefficient of the substance between two immiscible liquids. Nonpolar hormones are typically extracted from organic solvents. However, LLE is ineffective for all hormones exhibiting different polarities and often yields unsatisfactory efficiency. Various modifications and innovative techniques are being developed to overcome this problem.^129^ Moreover, modern LLE procedures assume sustainability in accord with green chemistry principles.

In the work of Fredj et al.,^130^ the use of LLE for the extraction of hormones from environmental samples was demonstrated for E1, E2, and EE2, which were investigated in Milli-Q water, tap water, and secondary treated wastewater samples. Decamethylcyclopentasiloxane (D5), being a substance not immiscible with water, was used as the solvent toward which the hormones were transferred. This study showed that LLE utilizing D5 is an effective method of extracting estrogens, even from wastewater. Moreover, the solvent was successfully recovered by gravity separation, so that it could be reused for extraction. Thus, the proposed approach is not only a suitable technique for sex hormone extraction from wastewater but also can be applied in water treatment for environmental protection purposes.

Another interesting environmentally friendly alternative approach for sample preparation in the analysis of hormone contaminants in water samples was developed using an aqueous two-phase system (ATPS).^131^ The ATPS-based method takes advantage of the two immiscible aqueous phases formed when certain polymers or salts are added to a solution, creating a system in which different biomolecules are partitioned between the two phases according to their physicochemical properties.^132^ ATPS consists primarily of water and additionally comprises various combinations of components that are generally known for low toxicity and affordability, such as polymer–polymer, salt–salt, polymer-salt, surfactant-salt, and polymer–surfactant. Domingues et al.^131^ used aqueous polymer solutions of PEO 1500 (HO(C_2_H_4_O)_*n*_H; MM = 1500 g/mol), L64 ((EO)_13_(PO)_30_(EO)_13_; MM = 2900 g/mol), and L35 ((EO)_11_(PO)_16_(EO)_11_); MM = 1900 g/mol) and aqueous salt solutions (Na_2_SO_4_, Li_2_SO_4_·H_2_O, MgSO_4_·7H_2_O, Na_2_C_4_H_4_O_6_·2H_2_O, Na_3_C_6_H_5_O_7_·2H_2_O). In those studies, particular attention was paid to the nature of the electrolyte and the forming polymer as well as parameters such as pH, tie-line length, temperature of the system, and mass ratio of both phases. High values of the partition coefficient were achieved regardless of the complexity of the matrix of the real water samples (drinking water, samples from lake and river water, and filtered water from a water treatment plant). Thus, the use of ATPS for the liquid–liquid extraction of steroid hormones proved to be efficient and cost-effective.^131^

Another approach is liquid-phase microextraction (LPME). It is a miniaturized version of traditional LLE and is considered to be more ecological, because of the reduced usage of chemicals. It provides a higher extraction efficiency than conventional LLE with shorter analysis times.^127^ Shi et al.^133^ used the combination of countercurrent salting-out homogeneous liquid–liquid extraction with dispersive liquid–liquid microextraction (CCSHLLE–DLLME) to determine estrogens in various environmental water samples by HPLC-UV. For this purpose, bottled water, river water, well water, and drinking water were collected as real samples. First, they were filtered through a 0.45 μm membrane filter to remove the suspended particles. Solvents such as MeOH and CCl_4_ were used for this method but in small volumes. Hormones were determined with recoveries ranging from 81.0 to 105.9% with detection limits of 0.09–0.20 ng/mL. Notably, this study presents the first application of CCSHLLE–DLLME in the determination of estrogens in environmental samples. In addition, it resulted in good sample throughput.^133^

Beldean-Galea et al.^134^ used a dispersive liquid–liquid microextraction method based on organic floating drop solidification to extract three estrogens (17β-estradiol, 17α-ethynylestradiol, and estriol) and four nonsteroidal anti-inflammatory drugs from wastewater samples. The method was described as easy to use. It distinguished itself by small sample volumes (10 mL) and required only 50 μL of low-toxicity extraction solvent (undecanol and acetonitrile). The obtained results showed extraction recoveries exceeding 80% for all compounds tested, except for estradiol, for which recovery ranged from 72.05 to 78.37%. The analysis did not show any considerable sensitivity to matrix effects.

Goh et al.^135^ presented liquid phase microextraction (LPME) using hollow fiber bundles for the determination of E1, E2, EE2, and E3 by UHPLC–MS/MS. The parameters influencing the extraction efficiency were examined. They included the number of hollow fiber bundles, the type of extraction, the applied desorption solvent, the mode of mixing, the temperature and duration of the extraction, and the salting-out effect. The developed method was characterized by high sensitivity and repeatability, and under the most favorable conditions, the detection limits were low, i.e., LODs between 0.251 and 0.440 ng/L and LOQs between 0.995 and 1.82 ng/L. The corresponding enrichment factors for the compounds tested ranged from 77- to 137-fold. The developed method was applied to wastewaters, and no significant matrix interferences were found.

Spray-assisted liquid-phase microextraction based on droplet formation (SADF-LPME) coupled with a GC-MS system was recently used by Zaman et al.^136^ In that study, hormones and pesticides were studied in rock, soil, water, moss, and penguin and seal feces samples collected from the Antarctic region. A mixture of dichloromethane:1,2-dichloroethane (1:1,v/v) was used as the extraction solvent. The authors focused on the optimization of the extraction process, including the type and ratio of extraction solvent, solvent volume, centrifugation period, and sample pH. The detection limit ranged from 1.0 to 6.6 ng/g, the limit of quantification ranged from 3.2 to 22.1 ng/g, and the enhancement factor ranged from 3.7 to 158.9. All of the obtained recoveries exceeded 70%. The satisfactory performance of the proposed method with various environmental samples suggests that it is a robust and straightforward approach for monitoring and studying organic pollutants (including steroid hormones) in analytical laboratory practice.

Recently, microextraction methods have also been modified to focus exclusively on environmentally friendly solvents such as ionic liquids (ILs)^137^ or deep eutectic solvents (DESs).^138,139^ Berton et al.^137^ determined the hormone content using extraction with ILs and then HPLC-UV. They used magnetic ionic liquids (MILs) to extract four estrogens, i.e., E1, E2, E3, and EE2, from environmental water samples (lake and sewage water). Trihexyl(tetradecyl)phosphonium cation ([P_66614_]^+^), to make MIL hydrophobic, was combined with tetrachloroferrate(III), ferrocyanide, and dysprosium thiocyanate, respectively, to obtain 3 ionic liquids ([P_66614_][FeCl_4_]), ([P_66614_ ]_3_[Fe(CN)_6_]), and ([P_66614_]_5_[Dy(SCN)_8_]). The use of magnetic counteranions simplifies extraction by preventing emulsion formation and eliminating the need for centrifugation. The method showed good repeatability and detection limits. Moreover, compared to solid-phase microextraction techniques, it takes comparable to or even less time.

The use of DESs, alone or combined with hollow fibers, nanofluids, or magnetic materials, shows promise for extracting EDCs, offering high recoveries and low detection limits. For example, Fattahi et al.^139^ introduced a new DES-LPME procedure coupled with GC-MS for the extraction and preconcentration of estrogenic compounds in environmental water and wastewater samples. A pH-switched hydrophobic DES was synthesized using *l*-menthol and (1*S*)-(+)-camphor-10-sulfonic acid (CSA). The limits of detection were in the range of 0.2 to 1.0 ng/L, and the recoveries of analytes (E1, E2, EE2, and E3) from environmental water and wastewater samples ranged from 91.0 to 108.8%.

#### Other Preconcentration Methods

Immunoaffinity extraction is a highly selective method for separating specific molecules such as hormones from complex matrices, such as biological samples but also environmental water samples. Both estrogens and progestogens, considered in this review, are biologically active substances that exhibit high specificity for binding to their antibodies. The target–antibody interactions lay behind the effectiveness of sex hormone immunoaffinity extraction ensuring minimal interference from other substances present in environmental waters. As an example, a simple, inexpensive, reusable, and environmentally friendly sample preparation method has been developed for the determination of estrogens.^61^ It used immunoaffinity SPME, a new version of immunoaffinity chromatography (IAC), consisting of antibodies immobilized on solid supports (fused silica fiber and fused silica capillaries). The IA-SPME rods applied as selective extraction sorbents prior to UPLC-MS/MS analysis allowed for the determination of trace amounts of estrogens in environmental water samples with LOD of 0.05–0.15 ng/mL and average recoveries from 34.2 to 62.7%.

Quick, Easy, Cheap, Effective, Rugged, and Safe extraction (QuECh-ERS) is a sample preparation method for the extraction of drugs, contaminants, pesticides, and hormones in environmental samples. It was first developed for food matrices; however, it has been later adapted for environmental applications with soil, water, sediment, and plant material,^62^ which makes it useful for screening aimed at potential risk assessment in aquatic toxicology. Recently, the QuEChERS extraction technique has been coupled with UHPLC-QTOF-MS to detect and quantify progestins (progesterone, desogestrel, drospirenone, gestodene, and levonorgestrel) in environmental water samples.^62^ In turn, in the study of Yang et al.,^140^ a method based on QuEChERS extraction and UHPLC-MS/MS was developed for the determination of estrogens and progestogens in fish and water. In another application of QuChERS, LC-TOF-MS was employed for the quantitative analysis of EDCs.^141^ This method achieved appropriate concentration levels for quantifying a wide range of compounds, including sex hormones and other pharmaceuticals, in sewage sludge samples, with detection limits between 1 and 2500 ng/g depending on the specific compound.

Durak et al. conducted a study using switchable solvent-based liquid phase microextraction (SS-LPME) and QuEChERS to determine organochlorine pesticides and hormones in environmental samples by GC-MS. This was the first report on SS-LPME and QuEChERS applications in determining these compounds. The method proved its suitability for analysis with high sensitivity and accuracy^142^ in quite demanding matrices (tap water, well water, lake water, medical wastewater, and tea samples). What exceeds the merit of this work but emphasizes the notable extractive capabilities of QuEChERS coupled with other preconcentration methods is the recent work of,^143^ where the authors applied QuEChERS and SPE for the determination of selected EDCs in soil samples. Namely, the combination of QuEChERS and SPE effectively removes matrix interferences and upgrades compound isolation allowing for high detectability at the simultaneous determination of a wide range of compounds including testosterone, progesterone, and estrogens (αE2, E2, E1, E3, EE2).^143^ The above examples indicate that the quick, easy, cheap, effective, rugged, and safe method is practical for hormone analytics in various environmental samples. Combining QuEChERS with other techniques can further enhance the sensitivity and accuracy of these analyses.

Another approach to sample preconcentration before hormone determination in environmental waters and wastewaters is the use of biochar-based membranes as sorption phases in thin-film microextraction. In the publication by Merlo et al., biochar derived from wood (Nuchar) was applied to a cellulose-based polymer matrix.^30^ The sorbent was used to extract hormones (including E1, E2, EE2, P, medroxyprogesterone acetate, hydroxyprogesterone, TST, and Epi-TST) from river water samples and the samples from WWTP. The later application of HPLC-MS/MS for qualitative and quantitative determinations proved the high efficiency of the proposed method.

Another membrane-based extraction technique, i.e., thin-film microextraction using polymer inclusion membranes, has been used by Merlo et al.^31^ Polymeric membranes turned out to be an effective and reliable sorptive phase for the simultaneous concentration of E2, EE2, E1, P, medroxyprogesterone acetate, and hydroxyprogesterone in a wide range of real water samples with good time efficiency, low solvent consumption, and good reproducibility. The studies provided a general picture of water contamination by endocrine disruptors in northern Italy.

An interesting approach combining bar adsorptive microextraction (BAμE) and microliquid desorption (μLD) has been proposed by Almeida et al.^18^ The introduced miniaturized BAE-μLD/HPLC–DAD device was used for the determination of nine steroid sex hormones (E1, E2, E3, αE2, EE2, P, 19-northisterone, d-(−)-norgestrel, and mestranol) in real water and urine matrices and was characterized by a good analytical performance: detection limits 50.0–100.0 ng/L, quantification limits 165.0–330.0 ng/L, linear dynamic ranges 0.2–24.0 μg/L, and intraday (RSD < 14%) and interday (RSD < 12%) repeatability.

### Derivatization

Some analytical methods for hormone determination require sample derivatization, with a notable example of GC-MS^20,27^ and related techniques (e.g., high-resolution gas chromatography with negative chemical ionization mass spectrometric detection HRGC-(NCI)-MS^144^) requiring hormone solution volatilization, reduction of polarity, and increasing thermal stability. The most common reagents for sample derivatization before GC-MS (also in context of hormone determination) are BSTFA (N,O-Bis(trimethylsilyl)trifluoroacetamide) in combination with a small amount of TMCS (trimethylchlorosilane).^20^ The parameters for optimal derivatization like BSTFA:TMCS proportion, the addition of other reagents (e.g., petroleum^27^), application of physical stimuli (e.g., microwave radiation^27^), temperature, heating time, and equilibration time, are analyte-dependent and require experimental verification.^20^

Sample derivatization can be also beneficial in the analysis of sex hormones by LC-MS.^28,145^ When the phenolic estrogens were derivatized with dansyl chloride, the sensitivity of the LC-MS/MS determination increased by one or two magnitudes for different analytes in comparison to the nonderivatized samples.^28^ Thus, derivatization may be a prerequisite for highly sensitive and accurate hormone determination; however, it can complicate workflows.

## Sex Hormone Determination

The detection methods of estrogens and progestogens in environmental waters can be divided into three main types: chemical analysis, immunoassays, and bioassays. These approaches substantially differ by their working principle, advantages, and limitations. Therefore, their usability significantly depends on the assumed aim of the hormone determination.

### Analytical Chemistry Approaches

Analytical chemistry approaches encompass both chromatographic and electrochemical methods. They enable reliable qualitative and quantitative determination of sex hormones, including simultaneous quantification of numerous derivatives and structurally similar molecules, with chromatography generally providing superior performance.

#### Chromatographic Analysis

In the context of estrogens and progestogens determination, chromatographic techniques mostly refer to the combination of chromatography (GC, LC, HPLC, UPLC) and mass spectrometry, with seemingly LC-MS/MS the most employed analytical approach to quantify sex hormones in aqueous environmental samples. The chromatographic approaches enable the reliable identification of sex hormones present in multicomponent mixtures and their accurate quantification.^82^ High efficiency, sensitivity, and selectivity offered by these techniques frequently outweigh their disadvantages, including high instrumentation costs, the need for operational expertise, relatively long analysis times, the use of organic solvents in liquid-based variants, and the potential need for sample derivatization to enhance volatility and detectability in GC, which can increase the processing time and analyte loss. Researchers have successfully applied chromatographic techniques to sex hormone analysis in environmental samples for years. In this work, we restrict ourselves to the most influential studies from the last ten years (see Table 2); hence, the earlier ones have been exhaustively outlined in literature (E2 and EE2 analytics,^81^ E1, E2, E3, EE2 determination,^146^ progestogens determination,^147^ analytics of androgens, estrogens, and progestogens^148^).

**Table 2:** Overview of methods determination of estrogens and progestogens in environmental aqueous matrices (2014–2024)

Matrix (Country)	Sample pretraetment	Analytical method	Recovery	LOD/LOQ	ref.
Wastewater (influent and effluent)	Filtration: glass fiber; Acidification: pH 3–4; **SPE**: Oasis HLB; Analyte elution: methanol; Derivatization: BSTFA, 1% TMCS, 65 °C, 1h	**GC**×GC–MS, EI; BPX 50 (30 m × 0.25 mm × 0.50 mm); BPX 1 (2 m × 0.1 mm × 0.1 mm)	89–98% (E1), 90—94% (E2), 87–97% (E3), 88–94% (EE2)	1.7–2.0 ng/L (E2), 6.7–8.6 ng/L (EE2)	(15)
Drinking water, wastewater	Filtration: cellulose filters, 0.45 μm; Addition: 1% formaldehyde; Centrifugation: 9400 rpm/30 min, 4 °C; **SPE**: C18 disk or cartridges; Analyte elution: ACN	**HPLC-DAD** (200 nm, 242 nm); Agilent Eclipse XDB C18 (250 × 4.6 mm, 5 μm); Gradient elution A: H_2_O/ACN (90:10 v/v) B: ACN	81–93% (E2), 82–96% (EE2)	6.3–9.0 ng/L (E2), 5.3–7.3 ng/L (EE2)	(14)
		**HPLC-FLD** (200 nm, 315 nm); Agilent Eclipse C18 (100 × 4.6 mm; 3.5 μm) Gradient elution: H_2_O/MeOH		2.1–7.5 ng/L (E2), 1.0–6.3 ng/L (EE2)	
		**HPLC–MS/MS, EI±** negative ionization mode Agilent Eclipse C18 (100 × 4.6 mm; 3.5 μm) Gradient elution: H_2_O/MeOH		0.16–0.21 ng/L (E2) 0.16–0.17 ng/L (EE2)	
Wastewater, groundwater, drinking water, river water	Filtration: filter paper, 11 μm; nylon, 0.45 μm; Ultrasonic degassing; FPSE, sol–gel poly-THF, coated extraction media; Analyte elution: methanol; Centrifugation: 5 min	**HPLC-FD** Supelco Acentis Express (150 × 4.6 mm; 5 μm); Gradient elution: ACN/MeOH/H2O (30:15:55, v/v)	89.4–97.4% (E2), 89.0–98.0% (EE2)	20 pg/mL (E2), 36 pg/mL (EE2)	(16)
Mineral water, runoff, wastewater	Filtration: polyester, 0.2 μm, Acidification: pH3, NaCl addition (30%, w/v); **DLLME**: CHCl_3_ (extraction solv.) ACN (dispersive solv.)	**MEKC-MS, ESI+** fused silica capillary (60 cm length, 50 μm i.d., 375 μm o.d.)	56–91% (E2), 57–79% (EE2), 53–68% (E3)	LOD: 0.35–0.92 μg/L (E2), 0.41–0.91 μg/L (EE2), 0.68–1.10 μg/L (E3)	(13)
River water (USA)	**small-scale LLE** in 1 mL of 2:5 methanol:MTBE, 1s, two extractions; Derivatization with dansyl chloride	**HPLC-MS/MS** Poroshell 120 EC-C18 (100 × 3.0 mm; 2.7 μm); Gradient elution A: water, B: acetonitrile		LOD: 0.045 ng/L (E1), 0.086 ng/L (E2), 0.030 ng/L (E3), 0.049 ng/L (EE2), 0.13 ng/L (equilin)	(145)
Freshwater (Lake Balaton) lakes and rivers	Filtration: Whatman glass microfiber filters; 0.45 μm; pH4; **SPE**: Strata C18-E, 55 μm, 70 Å, Analyte elution: MeOH	**HPLC-MS/MS, ESI+** Kinetex, C18 100 Å(100 × 2.1 mm; 2.6 μm), Gradient elution: A: 0.01% FA in H_2_O B: 0.01% FA in ACN	93.6–97.0% (P)	LOD: 0.003 ng/L (P), LOQ 0.03 ng/L (P)	(19)
River water (France, Belgium)	Filtration: Whatman glass microfiber, 45 μm; **SPE**: Oasis HLB, Analyte elution: MeOH/H_2_O (2:3, v/v), Derivatization: BSTFA, 1% TMCS, 65 °C, 2h	**GC-MS**	56–60% (E1), 51–55% (E2), 52–56% (E3), 50–54% (EE2), 66–70% (P)	LOD: 3.33 ng/L (E1, E2, E3, EE2), 1.66 ng/L (P); LOQ: 10 ng/L (E1, E2, E3, EE2), 5 ng/L (P)	(20)
Tap and bottled water (Poland)	**MIP-based SPE**	**HPLC/UV–vis** Lichrospher RP-C8 Symmetry (250 × 4.6 mm; 5 μm) MeOH:H_2_O (70:30, v/v)	32,5–96% (P)	LOD: 11.3 μg/mL (P); LOQ: 34.0 μg/mL (P)	(98)
River, wastewater (Switzerland)	Filtration: glass fiber membrane, 0.22 μm; **SPE**: Oasis HLB; Analyte elution A: ACN and ACN/EtOAc (1:1, v/v), Analyte elution B: 0.01–2% ammonia in MeOH	**LC-ESI-MS/MS, MRM** progestins analysis XSelect CSH C18 (100 × 3.0 mm; 2.5 μm) Gradient elution A: 0.5 mM HCO2NH4 and 0.1% HCOOH (v/v), B: MeOH; estrogens analysis XSelect CSH Phenyl-Hexyl (100 × 2.1 mm; 2.5 μm) Gradient elution A: 0.1 mM NH_4_F in H_2_O, B: MeOH in ACN (1:3, v/v)	82–120%	LOD: 0.01–40 ng/L, RSD: 1–13%	(21)
Raw and treated wastewater (Poland)	Acidification (with or without, pH3 or 6,5) **SPE - triple-sorbents** Strata X (Phenomenex)+PSA+Alumina; Analyte elution: MeOH; Derivatization: BSTFA, 1% TMCS, 60 °C, 0.5 h	GC/MS(SIM), EI RTX-5MS (30 m × 0.25 mm × 0.25 μm, Restek)	75–91% (E1), 78–96% (E2), 84–92% (TST), 94–112% (EE2), 82–106% (E3)	MDL/MQL: 17/50 ng/L (E1), 33/100 ng/L (E2), 33/100 (TST), 17/50 (EE2), 33/100 (E3), RSD: 0.8–8.2% (E1), 2.0–6.0% (E2), 5.8–7.8% (TST), 3.0–9.4% (EE2), 6.0–8.8% (E3)	(23)
real water samples (Poland)	Filtration: 0.45 μm nylon filter; **MISPE** (surface modification of spherical silica gel pore size 60–100 Å); Analyte elution: A: H_2_O; B: 5% MeOH	**HPLC/DAD** the homemade cholesterol-based SG-CHOLIC#1 column (150 × 4.6 mm) MeOH/H_2_O (53:47, v/v)	78.7–101.3%	LOD: 0.0182–0.0898 μg/mL, LOQ: 0.0551–0.272 μg/mL	(115)
plastic bottle, tap water and surface water	Filtration: 0.45 μm membrane; **in tube SPME**	**HPLC-DAD** Zorbax C18 column (250 × 4.6 mm; 5 μm); ACN: H_2_O (50:50, v/v)	80–129%;	LOD: 0.05–0.20 μg/L, RSD: < 2.5%	(88)
tap, surface, wastewater (Canada)	Filtration: glass fiber filters, GFF, 0.3 μm	**SPE-UHPLC-MS/MS, heated-ESI** Online SPE: two Hypersil Gold aQ C18 (20 × 2 mm; 12 μm) connected in series; Analytical: Hypersil Gold C18 (100 × 2.1 mm; 1.9 μm) A: water, B: 100% MeOH, C: 1 mM NH_4_F in H_2_O	70–130%	LOD: 0.05–1.0 ng/L, RSD: 1.3–19%	(22)
River water (Brazil)	Filtration: 0.45 μm celulose membrane; **EC-SPE** 1. steel mesh electrodes, PPy modified, a globular morphology 2. PPyNTs electrodes with nanotube morphology, Analyte elution: MeOH	**GC-MS** SH-Rtx-5MS (30 m × 0.25 mm × 0.25 μm - Shimadzu) 1.0 mL/min;	n.d.	LOD: 16.7 μg/L, RSD: 25.3–26.3% (P)	(106)
wastewater, river systems (South Africa)	**UA-DMSPE** 1. Fe_3_O_4_–Al_2_O_3_@CNFs nanocomposite synthesis 2. Nanosorbent washing (MeOH, H_2_O) and its dispersion into a sample solution (pH 4, ultrasonication, 30 min) 3. Sorbent separation from sample solution (magnet at the bottom) 4. Addition of ACN and ultrasonication (5 min) 5. Separation of adsorbent and analyte (an external magnet)	HPLC-DAD Column C18 Gradient elution: A: ACN/H_2_O (45:55, v/v)	93.0–99.3%	LOD: 0.009 μg/L (E2) LOQ: 0.03 μg/L (E2) RSD: 1.2—3.7% (E2)	(150)
surface wastewater (Argentina)	Filtration: Whatman nylon membrane filters, 45 μm; **SPE**: C18, Oasis HLB; Analyte elution: MeOH	**HPLC-MS/MS** Eclipse plus C18 (100 × 2.1 mm; 3.5 μm), A: 0.1 mM NH_4_F in H_2_O, B: 100% MeOH	42–144%	LOD/LOQ: 19–63 ng/L (E1), 32–106 ng/L (E2), 29–98 ng/L (EE2), 19–63 ng/L (E3), 2–8 ng/L (17OHP), 1.9–6 ng/L (P)	(24)
Wastewater (RPA)	Filtration: Whatman GF/F glass fiber filter, 0.7 μm; Acidification: HCl, pH3; **SPE**: Oasis HLB, Analyte elution: MeOH	**LC-MS/MS, ESI** InertSustain C18 column (150 × 2.1 mm; 3 μm), Linear gradient elution: A: 0.1% NH_4_OH in H_2_O, B: 0.1% NH_4_OH in MeOH	n.d.	LOD: 0.38–1.30 ng/L, LOQ: 1.71–2.41 ng/L	(152)
surface water (river, canal USA)	Filtration: FilterMateTM, PTFE, 0.2 μm	**online SPE-LC-HRMS APCI** Hypersil Gold aQ; SPE (20 × 2.1 mm; 12 μm), Analytical (100 × 2.1 mm; 1.9 μm), A: H_2_O, B: MeOH	96–101%	LOD: 0.2–10.5 ng/L, RSD: < 20%	(25)
drinking water, lake water, river water and filtered water from WTP	**ATPS-LLE** experimental condition for the recovery of all hormones simultaneously: L35 + Na2SO4 + H2O, pH = 3.0; TLL: 64.06% w/w, 298.15 K; mass ratio between the bottom and top phases of the system = 5 electrolyte and polymer preconcentration factor of the analytes: 7–44	UV–vis		K: 8.8–4533	(131)
Farms, wastewater treatment plants (China)	Filtration, pH3; **SPE** (Oasis HLB), Analyte elution: MeOH/DCM	**LC-MS/MS, ESI/MRM** Agilent Poroshell EC-C18, Gradient elution: A: ACN, B: 0.1% ammonium acetate in H_2_O	71–121%	LOQ: 0.004–0.03 ng/L (PG)	(26)
Surface, wastewater, tap, mineral, well, pond, swimming pool, river, waste (Spain)	Filtration: cellulose filters, 0.45 μm; Samples adjusted to neutral pH (HCl or NaOH); **SPE**: Oasis HLB, Analyte elution: acetone; Derivatization: BSTFA, 1% TMCS petroleum ether, microwave oven, 200 W, 4 min	**GC-MS** DB-5 fused silica capillary column (30 m × 0.25 mm; 0,25 μm) coated with 5% phenylmethyl polysiloxane (Supelco)	92–103%	LOD: 0.01–0.30 ng/L, RSD: 3–7%	(27)
drinking water, mineral water (Hungary)	**SPE**: Envi-C18 DSK, Analyte elution: MeOH; Derivatization: 0.1 M Na_2_CO_3_, Dansyl chloride in ACN (60 °C, 10 min)	**HPLC- MS/MS, HESI+(PRM)** Waters XTerra MS C18 (150 mm × 2.1 mm; 3.5 μm), Gradient elution: A: 0,1% FA in H_2_O, B: 0,1% FA in ACN	89–114%	LOD: 10 pg/L	(28)
seawater (Rizhao, China)	**Membrane SPE** 1. Synthesis of sorbent: MIL-53(Al)/MMMs 2. Preconditioning of sorbent (vortexing, MeOH, H_2_O, 1 min) 3. Vortexing of sorbent and aqueous sample (30 min) 4. Removal of the sorbent (tweezers) 5. Desorption of the analytes (MeOH, fierce vortexing, 20 min) 6. Filtration (0.22 μm organic membrane)	**HPLC-FLD** TOSOH TSKGEL, ODS-100 V analytical column (150 × 4.6 mm; 3 μm), Gradient elution: H_2_O/ACN (45:55, v/v)	90.7—102.5%	LOD: 0.12 μg/L (E1), 0.005 μg/L (E2, E3), 0.009 μgg/L (EE2), LOQ: 0.5 μg/L (E1), 0.015 μg/L (E2, E3), 0.025 μg/L (EE2)	(153)
surface water	**LLE** ATPSs based on double PILs [2-HEA][Ac] + [BHEA][Ac]) + MeCN	**UHPLC** C-18 column (100 × 2 mm); Gradient elution A: H_2_O/ACN with 0.1% HCOOH, B: ACN		LOD: 2.8 ng/L (P), 2.7 ng/L (E2), 3.2 ng/L (E3), 4.1 ng/L (EE2), LOQ: 9.2 ng/L (P), 8.9 ng/L (E2), 10.5 ng/L (E3), 13.5 ng/L (EE2)	(29)
wastewater, tap water	**TF-ME** Sorbent: 3D MMM (graphitic carbon nitride/polyvinylpyrrolidone), pH 8	**HPLC** Ultisil XB-C18 column (250 × 4.6 mm; 5 mm), Elution: H_2_O/ACN (20:80, v/v)	Tap water: 100.86% (E1), 107.97% (E2), Wastewater: 99.72% (E1), 108.28% (E2)	LOD/LOQ: 5.0 μg/L (E1), 1.5 μg/L (E2)	(154)
River water, wastewaters (Italy)	**TF-ME** Sorbent: biochar-based polymeric film; Membrane: cellulose based polymeric matrix and a wood-derived biochar (10% w/w Nuchar); native pH 8 h; orbital shaking at 250 rpm/min; Analyte elution: EtOH, 5 min, 250 rpm/min	**HPLC-MS/MS (ESI±) (MRM)** Agilent Zorbax Eclipse Plus C18 (150 × 2.1 mm; 3.5 μm), Gradient elution: 1 mM NH_4_F in H_2_O/ACN	96–123% (E1), 65–118% (E2), 63–100% (E3), 64–119% (EE2), 68–94% (P), 73–128% (TST),	LOD: 0.1–0.5 ng/L (E1), 0.3–0.4 ng/L (E2), 0.2–0.3 ng/L (EE2), 0.1–0.4 ng/L (P), 0.1–0.3 ng/L (TST), LOQ: 0.2–1.6 ng/L (E1), 1.0–1.3 ng/L (E2), 0.5–1.0 ng/L (EE2), 0.4–0.7 ng/L (P), 0.2–0.8 ng/L (TST),	(30)
Portuguese Estuaries (aquatic ecosystems)	**QuEChERS** freeze-dried matrices (bivalve, polychaete, crustacean); Add: a) distilled water and ACN, *n*-hexane, b) a mixture: sodium citrate, disodium citrate, sodium chloride, magnesium sulfate; Vigorous vortexing and centrifugation; Filtration: PTFE 13 mm	**UHPLC-QTOF-MS/MS** Acclaim 120 C18 (100 × 2.1 mm; 2.2 μm), Gradient elution: A: H_2_O with 0.1% HCOOH, B: MeOH with 0.1% HCOOH	75.5 ± 1.26 (Drospirenone), 72.70 ± 7.17 (Gestodene), 96.40 ± 6.23 (Levonorgestrel)	LOD: 0.16 ng/L (Drospirenone), 0.15 ng/L (Gestodene), 0.08 ng/L (Levonorgestrel), LOQ: 0.48 ng/L (Drospirenone), 0.45 ng/L (Gestodene), 0.24 ng/L (Levonorgestrel)	(62)

Considering the coupling of a chromatographic setup with different types of analyzers, the determination efficiency for E1, E2, E3, EE2, and P in drinking water and wastewater samples was investigated using HPLC coupled with diode array detection, fluorescence detection, and tandem spectrometry.^14^ The results proved HPLC-MS/MS to be the most sensitive and precise analytical approach of the inspected ones. The high sensitivity of various mass spectrometers and their broad applicability in the context of reliable detection of sex steroid hormones from aqueous matrices is confirmed by many studies (see Table 2). At the same time, the advantages and disadvantages of different MS types have been nicely summarized by Song and Feng in an article,^84^ which only omits some latest advancements (like, e.g., the recent advances in high-resolution laser desorption/ionization mass spectrometry (LDI-MS) and mass spectrometry imaging^149^). Still, however, some authors decide to use some other detector types, like UV–vis^98,131^ reaching good LOD at the level of tens of μg/mL, DAD^88,115,150^ allowing to attain more satisfactory detection limits (even several ng/mL), FD^16^ giving LOD at the level of tens pg/mL, which is still at least 1 order of magnitude worse than the typical sensitivity of a well-optimized chromatographic system coupled to mass spectrometry.

The possible improvements in chromatographic analysis of hormones are related to the development of faster, simpler, and greener methods for sensitive simultaneous determination of multiple analytes at ultratrace levels. Considering works where particularly high sensitivity has been attained, in reports,^19,26,28^ the applied analytical procedures enabled for reaching picograms per liter limit. The chromatographic analysis is particularly well-suited to the simultaneous analysis of multiple analytes, as shown, e.g., for progestins: levonorgestrel, drospirenone, and progesterone by HPLC-MS/MS,^19^ 62 progestins (including, among others, P, 20α-dihydroprogesterone, 20β-hydroxyprogesterone, 17α-hydroxyprogesterone),^87,151^ drospirenone, gestodene, levonorgestrel by UHPLC-QTOF-MS/MS,^62^ estrogens: E1, E2, E3, EE2 by LC–MS/MS,^17^ E1, αE2, E2, E3, EE2 by GC × GC-qMS,^15^ E1, E2, E3, EE2, equilin by HPLC-MS/MS,^145^ E1, E2, E3, EE2 by LC-MS/MS,^152^ E1, E2, E3 by GC-MS,^105^ hormone mixtures: E1, E2, E3, EE2, P by HPLC-MS/MS,^14^ 13 EDCs (including P, E1, E2, E3, EE2, TST) by GC-MS,^20^ 2 progestin metabolites (17α-hydroxypregnanolone and pregnanediol) and 31 different steroids (including E1, E2, E3, P, TST) by HPLC-MS/MS,^21^ E1, E2, E3, EE2, P, and TST by HPLC-DAD,^115^ E1, E3, EE2, BPA, diethylstillbestrol, hexestrol by HPLC-DAD,^88^ 14 EDCs (TST, P, medroxyprogesterone, levonorgestrel, norethin-drone, androstenedione, estrone, α-estradiol, β-estradiol, equilin, equilenin, ethinylestradiol, estriol, BPA) by UHPLC-MS/MS,^22^ E1, E2, E3, EE2, P, 17α-hydroxyprogesterone, TST, DHT by HPLC-MS/MS,^24^ 11 EDCs (including E1, E2, E3, EE2, and equilin) by LC-HRMS,^25^ E1, E2, EE2, hexestrol, P, TST, and DHT by GC-MS,^27^ E1, E2, E3, EE2, TST, and androstenedione by LC-MS/MS,^28^ 13 sex hormones (estrogens, progestins, androgens, and prostaglandins) using LC-MS/MS,^75^ EDCs (including E1, E2, EE2, P, medroxyprogesterone acetate, hydroxyprogesterone, TST, Epi-TST) by HPLC-MS/MS,^30^ or 25 EDCs by LS-MS/MS.^59^

Considering the sustainability of analytical methods, including minimization or elimination of organic solvent use, GC methods emerge as environmentally friendly due to the usage of an inert gas as the mobile phase. Another, quite untypical eco-friendly approach from the chromatographic analytics family, was presented by D’Orazio et al.,^13^ where 12 estrogenic compounds have been quantified by application of dispersive liquid–liquid microextraction and micellar electrokinetic chromatography (MEKC) coupled to electrospray ion trap mass spectrometry. This analytical strategy distinguished itself by very low consumption of reagents and samples and affordable operational expenses at the cost of sensitivity reduction. Still, MEKC can be considered a strong alternative to the popular LC-MS approach, especially from the perspective of the green chemistry assumptions.

#### Electrochemical Analysis

Frequently, the first-choice method for sex-hormone determination is liquid chromatography coupled with tandem mass spectrometry both in the clinical practice,^155^ environmental monitoring,^22,156^ and dedicated research, especially, when simultaneous determination of several different hormones is needed.^21,87,91^ Notwithstanding, because the electrochemical analysis frequently offers advantages like low detection limit, fast detection speed, high reproducibility, simplicity, minimizing (or even skipping) sample preparation stage, low signal burden by matrix effects (at least in terms of aqueous environmental samples), and relative ease of miniaturization (which fosters the development of quick testers and marketable point-of-care devices for noninvasive hormone monitoring), they have gained a considerable scientific and commercial interest.^157,158^

Electrochemical sensors offer another possibility of reliable sex hormone determination in aqueous environmental samples.^157,158^ The basic principle that underlies electrochemical sensing is that a hormone of interest should be directly oxidized or reduced at the electrode surface, resulting in changes in the electrochemical properties of the analyzed solution, including electric potential, conductance, and current, which can be measured by electrochemical techniques. Among the popular techniques utilized in electrochemical hormone sensing, one can list single-potential amperometry, pulsed-amperometry, cyclic voltammetry (CV), differential pulse voltammetry (DPV), square wave voltammetry (SWV), and electrochemical impedance spectroscopy (EIS).

In recent years, the electrochemical analytics of sex hormones underwent substantial progress due to the deployment of advanced electrode platforms, which allowed to overcome typical methodological pitfalls including sensitivity to matrix effects, weak long-term stability, susceptibility to the influence of physicochemical factors (temperature, pressure, electromagnetic field), or liability to contamination of electrode surface (that impairs charge transfer). In particular, the overall performance of estrogens and progestegens determination by electrochemical methods has been easily improved by including advanced electrodes from noble metals (e.g., gold^159,160^) or fabricating advanced hybrid electrodes (e.g., modified glassy carbon electrode like carbon dots-polyaniline/GCE,^161^ graphene quantum dots and poly(sulfosalicylic acid) comodified electrode,^162^ carbon paste electrode modified by Au nanoparticles on bimetallic metal–organic framework AuNPs@AuZn-MOF/CPE^163^). Such complex electrode systems improve the conductivity, increase the electrochemically active surface area, and augment catalytic performance. Another promising direction, that enabled advancements in the construction of electrochemical sex hormone sensors applicable in the analysis of aqueous environmental samples, is the modern adaptation of existing technologies to construct electrodes (like screen-printing with customized conductive inks^164−168^). In turn, the relatively high selectivity of hormone detection can be ensured by applying tailored sensitive receptor systems for the determined hormones (e.g., by ’functional’ coating with bioreceptors, as described in detail in the section Biosensors) or applying molecularly imprinted polymers^111,157,169−171^). Furthermore, enhancement of surface area and improvement of its electrochemical characteristics are also beneficial. It provides fast mass transfer, low interfacial resistance, and low ohmic drop at the electrode surface due to radial diffusion and consequently leads to a notable signal amplification, which enables detection limit minimization. This can be reached via the construction of complex electrode frameworks or modifying electrode interface, e.g., by employment of advanced electrochemical core–shell nanosystems like nanoparticles coated with molecularly imprinted conducting polymers. Depending on the requirements of the developed biosensing electrochemical platform, nanomaterials of different dimensions can be incorporated as electrode surface modification, including 1D (e.g., nanotubes, nanowires, nanorods), 2D (e.g., graphene, nanosheets), and 3D (e.g., nanoparticles, quantum dots) nanomaterials.^157^ In the further part of this section, we discuss in a more detailed way the selected aspects of the recent progress in the construction and performance of electrochemical nanosensors for estrogen and progestogen detection with appropriate examples, which we finally outline in Table 3.

**Table 3:** Summary of prominent representatives of electrochemical sensors of estrogens and progestagens used in the analysis of environmental waters (and some selected aqueous samples of similar relatively simple matrices)

Hormone	Strategy for electrode modification	Electrochem. method	Sample type	LOD/LOQ, M	Linear range, M	ref.
E2	CDs-PANI/GCE (GCE modified by polyaniline and carbon dots))	CV, LSV	drinkable, tap, and river water	4.3 × 10^–8^	10^–9^–10^–4^	(161)
E1	AuNPs@AuZn-MOF/CPE (carbon paste electrode modified by Au nanoparticles on bimetallic metal–organic framework)	DPV	lake water	1.23 × 10^–8^	5 × 10^–8^–5 × 10^–6^	(163)
EE2	(mag@MIP)-GQDs-FG-NF/SPE (screen-printed electrode modified with functionalized graphene, graphene quantum dots and magnetic nanoparticles coated with molecularly imprinted polymer)	SWV	river water	2.6 × 10^–9^	10 × 10^–9^–2.5 × 10^–6^	(164)
E2	MIP-mag/SPCE (screen printed carbon electrode modified by Fe_3_O_4_-MIP (E2-MIP PANI))	SWV	river water	2 × 10^–8^	5 × 10^–8^–10^–6^	(165)
E2	rGO-AuNPs/CNT/SPE (screen-printed carbon electrode modified by gold-nanoparticle-decorated reduced graphene oxide-carbon nanotubes)	DPV	drinkable water	3 × 10^–9^	5 × 10^–8^–10 × 10^–6^	(166)
E2	CuPc-P6LC-Nafion/SPCE (screen printed carbon electrode modified by CuPc, P6LC and a Nafion film)	DPV	envir. waters	5 × 10^–9^	8 × 10^–8^ – 7.3 × 10^–6^	(167)
P	AuNPs-(5-Amino-2-mercaptobenzimidazole)-rGO/SPCE (screen printed carbon electrode modified by reduced graphene oxide, 5-Amino-2-mercaptobenzimidazole, gold nanoparticles)	SWV	P in its aqueous basic ethanol solution	2.8 × 10^–10^	0.9 × 10^–9^–2.7 × 10^–5^	(168)
E2	MIP-PB-MIL-CNTs/GCE (GCE modified by metal–organic frameworks, carbon nanotubes, Prussian blue and E2-MIP (polypyrrole))	DPV	pond water	6.19 × 10^–15^	10^–14^–10^–10^	(172)
E1	Ru(bpy)_3_^2+^/MWCNTs/Nafion/Au Au composite electrode based on E1-MIP, Ru-bpy luminescent reagent, multi walled carbon nanotubes and Nafion)	ECL	river water	1.7 × 10^–11^	3.7 × 10^–10^–7.4 × 10^–7^	(160)
E2	MIP-CB/CPB (Carbon paste electrode modified with carbon black and E2-MIP)	DPAdSV	river water	3 × 10^–8^	10^–7^–2.3^–5^	(173)
E1, E2, E3, EE2	MIP/rGO/CPE (carbon paste electrode modified by reduced graphene oxide (rGO) and EE2-MIP)	DPAdSV	river water	2.7 × 10^–8^ for each hormone	1.6 × 10^–7^–1.5 × 10^–5^ for each hormone	(174)
E2	nanoMIP-doped PANI/Au (electropolimerized polyaniline on Au electrode doped with E2-MIP nanoparticles)	EIS	waste- water	2.86 × 10^–12^	3.67 × 10^–11^–3.67 × 10^–8^	(175)
E2	MIPs-AuNP (E2-MIP/Au (polythioaniline)-functionalized AuNPs on gold electrode)	LSV	river water	1.09 × 10^–15^	3.6 × 10^–15^–3.6 × 10^–9^	(159)
E2	MIP-nanowell Au film/Au (E2-MIP (polythioaniline) on nanowell Au film immobilized on Au electrode)	CV	drinkable water	10^–13^	10^–12^–10^–5^	(176)
E3	MIP NPs/GCE (GCE modified by micelle assisted electroactive E3-MIP polypyrrole NPs)	DPV	aqueous samples (H_2_O:ACN 1:1 v/v)	1.6 × 10^–7^	5 × 10^–5^—5 × 10^–6^ and 10 × 10^–5^—10 × 10^–4^	(177)
E2	MIPs-PtNP/GCE (6-mercaptonicotinic acid polymerized on the surface of platinum nanoparticles-modified glassy carbon electrode)	DPV	wastewater (before and after threatment) and tap water	1.6 × 10^–8^	3.0 × 10^–8^–5.0 × 10^–5^	(178)
E2	MIPs-AgNP- (E2-MIP poly(*p*-aminophenol) supported by silver nanoparticles capped with 2-mercaptobenzoxazole)	CV, SWV	real water samples	1.86 × 10^–12^	10^–12^–10^–9^	(179)
E2	Fe_3_O_4_-MIP@RGO (E2-MIP sensor based on Fe_3_O_4_ nanobeads immobilized on graphene)	DPV	water solution	0.8 × 10^–9^	5 × 10^–8^–10^–5^	(180)
E2	α-Fe_2_O_3_–CNT/GCE (glassy carbon electrode modified by α-Fe_2_O_3_ nanoparticles and carbon nanotubes)	SWV	lake water	4.4 × 10^–9^	5 × 10^–9^–10^–7^	(181)
P	GO-IMZ/GCE (GCE modified by imidazole-functionalized graphene oxide)	SWV	P water solution	6.8 × 10^–10^	2.2 × 10^–7^–1.4 × 10^–5^	(182)
E3	rGO/AgNPs/GCE (GCE modified by reduced graphene oxide and Ag nanopartices)	DPV	tap water	2.1 × 10^–8^	10^–7^–3 × 10^–6^	(183)
E3	rGO–SbNPs/GCE (GCE modified by reduced graphene oxide and Sb nanopartices)	DPV	natural water sample (dam reservoir)	5 × 10^–10^	2 × 10^–7^–1.4 × 10^–6^	(184)

One of the most promising approaches is to apply MIP-based sensors. As mentioned before in subsection *Solid Phase Extraction*, the molecularly imprinted polymers can function as artificial bioreceptors, which establish high selectivity and sensitivity due to the customizable binding sites for considered hormone targets, high stability (also in harsh conditions), and cost-effectiveness.^111,169−171^ On the contrary, among the possible drawbacks one can mention the importance of optimizing the polymer thickness, especially for the nonconductive polymers. (An increase in the MIP layer thickness contributes to an elevation in the electrical resistance, but contrastingly it also leads to an increase in the number of selective sites available for hormone binding. Thus, a trade-off between these two effects has to be reached.) Considering other recurring problems of MIP-based electrochemical sensors, frequently, there can occur limitations in terms of sensitivity, power output, and reproducibility.^157^ To increase the effective capacity of the imprinted polymer film, frequently advanced materials (e.g., metal–organic frameworks and nanoparticles) are combined with MIP, which allows one to achieve enhanced performance of hormone sensing via functional synergy. Over the course of time, a plethora of MIP-based electrochemical sensors of estrogens and progestogens have been proposed, which can be successfully applied in environmental water analysis. Here we outline some selected studies that highlight interesting prospects for the field.

An E2-sensitive hybrid sensor has been proposed by Duan et al.^172^ It was based on metal–organic frameworks (here MIL-53(Al), ensuring a porous 3D matrix), carbon nanotubes (to improve the electrical conductivity and increase effective surface area), Prussian blue (Fe_4_[Fe(CN)_6_]^3–^, to enhance electrocatalytic activity and redox properties), and E2-imprinted polypyrrole (for E2 sensing). The resulting novel MIP-PB-MIL-CNTs/GCE exhibited a wide linear response range of E2 concentration between 10^–14^ to 10^–9^ M and LOD = 6.1910 × 10^–15^.

An advanced MIP-based electrochemical sensor was developed using Ru(bpy)_3_^2+^/MWCNTs/Nafion/gold electrodes covered with E1-MIP for ultrasensitive estrone quantification in water samples via electrochemiluminescence (ECL) measurement.^160^ The obtained ECL sensor allowed detection of as low E1 concentrations as 0.0047 μg/L.

In turn, Da Silva and Pereira modified the carbon paste electrode with MIP (E2-imprinted; using methacrylic acid as a functional monomer) and carbon black (CB) for elevated surface area, electrical conductivity, and fast electron transfer kinetics. These modifications yielded an increased sensitivity of over 173% in relation to that of the bare electrode, wide linear range (0.10–23.0 μM), and low E2 detection limit (30 nM) in the study of river water samples.^173^ Another example of carbon paste electrode modification by MIPs with the aim of estrogens’ determination in environmental waters was proposed by Braga et al.^174^ This time, however, additionally reduced graphene oxide was used in electrode fabrication, and the EE2 was taken as a template molecule during molecular printing of MAA-derivative polymer. Moreover, the authors investigated the determination of multiple compounds—estrone, estradiol, ethinyl estradiol, and estriol—with a strong emphasis on specificity.^174^ The results of differential-pulse adsorptive stripping voltammetric (DPAdSV) measurements showed that the electrochemical behavior of the 4 analytes in question was similar. The DPV signals of analyzed estrogens have, however, substantially differed from those of phenolic compounds catechol and hydroquinone. For this reason, the introduced CPE/rGO/MIP sensor was said to be selective to estrogens and appropriate for total determination of the class of estrogenic compounds. Nonetheless, it did not reveal individual discernible analytical responses for E1, E2, EE2, and E3 when real samples were investigated. In this context, the study by Braga et al. highlights the inherent limitations in the specificity of electrochemical sensing, particularly when quantifying a group of structurally similar derivative compounds simultaneously.^174^ While this limitation may be acceptable in certain applications, such as evaluating the total estrogenic burden, it becomes a significant methodological drawback in broader contexts, where determining the individual contribution of each estrogen is essential.

A popular practical strategy in hormone analytics is to utilize MIP nanoparticles or MIP-based core–shell nanoparticles, which generally exhibit better electrochemical characteristics than plain MIP layer. Marinangeli et al. considered the determination of E2 in wastewater using electrochemical impedance spectroscopy and paid particular attention to the impact of the distribution of binding sites predetermined by the MIP synthesis procedure.^175^ Namely, they studied the effects of inhomogeneous versus homogeneous imprinted binding sites on electrochemical sensing measurements and the overall sensor performance. To find a practical representative of ”inhomogeneous binding sites”, working electrodes were modified with an E2-MIP polyaniline (PANI) thin layer, called the “Imprinted PANI layer”, whereas the ”homogeneous binding sites” were gained by electrode modification with a thin PANI layer purposedly doped with E2-MIP nanoparticles (nanoMIP-doped PANI). According to the results, homogeneity in the surface distribution of the binding sites ensures a high sensor sensitivity. The sensitivity of nanoMIP-doped PANI was almost twice as sensitive as inhomogeneous PANI and responded linearly to E2 concentrations from 36.7 pM to 36 7 nM with an LOD of 2.86 pg/mL.

Another interesting concept of modifying the working electrode with MIP-covered noble metal nanoparticles allowed one to avoid a complex sample pretreatment and reach a very low limit of hormone quantification. For example, the limit of 17β-estradiol quantification of 1.09 fM has been reached by the application of a voltametric sensor obtained by coating of gold working electrodes with MIP (polythioaniline) film manufactured by potentiodynamic electropolymerization of *p*-aminothiophenol on gold nanoparticles (AuNPs).^159^ Additionally, a related study introduced the nanowell gold film with a thickness of 120 nm and a pore size of 20 nm, immobilized on an Au electrode and covered with MIP, which served as the electrochemical E2 sensor.^176^ Notably, the aforementioned studies showed that MIP nanoparticles or MIP-covered nanoparticles can be held on electrode surfaces by electropolymerized thin films. Nevertheless, the integration of the analyte-sensitive nanoparticles after their synthesis over the transducer surface can be a challenging step. An interesting approach has been proposed, feasible at least for some polymers, where E3-MIP polypyrrole nanoparticles were synthesized with the aid of micelles.^177^ The fabricated chemosensor allowed for the determination of estriol in the concentration range of 0.5–5 μM and 10–100 μM with the 0.16 μM limit of detection (LOD). The concept of MIPs-(noble metal nanoparticles)/GCE system has also been applied in the study, where a MIP-based E2 sensor was developed through the electropolymerization of 6-mercaptonicotinic acid on Pt nanoparticles-modified GCE.^178^ The proposed sensor was able to detect concentrations of E2 as low as 1.6 × 10^–8^ M in water samples. In turn, molecularly imprinted poly(*p*-aminophenol) supported by silver nanoparticles capped with 2-mercaptobenzoxazole turned out to be an effective base for the E2 sensor with LOD = 1.86 nM.^179^

A robust subfamily of MIP-based sensors for sex hormones is magnetic molecularly imprinted polymers-based systems. They exhibit several superior characteristics related, among others, to a controllable rebinding process and permission for easy and quick magnetic separation. Magnetic molecularly imprinted polymers can be easily attracted to the surface of a magnetic electrode, which could simplify the process of electrode surface modification and improve the stability of the electrode.^164,165^ As a prominent example, a novel sensor for E2 in water samples was constructed using Fe_3_O_4_ nanobeads/reduced graphene oxide composite covered by MIP (with MAA as monomer) polymerized by a reversible addition–fragmentation chain transfer (RAFT) technique.^180^ Application of differential pulse voltammetry measurement with the obtained Fe_3_O_4_-MIP@RGO drop-casted on a polished glassy carbon electrode allowed for reaching the detection limit of 0.819 nM and a linear range from 0.05 to 10 μM.

Magnetic nanoparticles have been also introduced to the simpler electrochemical hormone sensors, being deprived of tailored hormone receptors (like MIPs). A modification of a glassy carbon electrode with a nanocomposite consisting of α-Fe_2_O_3_ nanoparticles supported on carbon nanotubes (CNTs) allowed for efficient E2 detection by SWV with LOD = 4.4 nM and linear concentration range of 5.0–100.0 nM.^181^

Researchers have explored the use of various carbonaceous materials, including graphene, graphene oxide, reduced graphene oxide, carbon dots, carbon black, carbon nanofibers, and carbon nanotubes, for the electrochemical sensing of estrogens and progestogens in aqueous environmental samples, as shown in numerous studies.^160,172−174,180,181^ Among the main benefits of the fabrication of composite carbonaceous electrodes are the possibility of easy structure functionalization, elevated electrical conductivity, and large surface area. An effective voltametric P sensor was constructed by modification of GCE by imidazole-functionalized graphene oxide (GO).^182^ GO can be described as a sheet based on hexagonal and planar graphene lattice with various oxygenated functional groups covalently bonded on its surface, which are responsible for the material hydrophilicity, and also make this material an attractive platform for covalent immobilization of molecules or interest groups (there - imidazole, which can effectively interact with progesterone molecules via π–π interactions). In turn, the reduced graphene oxide exhibits rGO higher electric conductivity than GO, but still it can be easily chemically functionalized due to its oxygenated residua and structural defects originating in the chemical oxidation synthesis of GO. Therefore, rGO has been used even more often in the construction of electrochemical hormone sensors. To illustrate, rGO together with metal nanoparticles has been used to construct effective estriol sensors.^183,184^ In particular, the proposed rGO-AgNPs/GCE was successfully applied to the determination of estriol in tap water in concentration regime 0.1 to 3 μM (LOD = 21 nM).^183^ In turn, the rGO–SbNPs/GCE was used for the determination of estriol in natural water.^184^ This device gave a linear response over the concentration range of 0.2 to 1.4 μM, with a detection limit of 0.5 nM.

Finally, one of the most distinctive advantages of electrochemical sex hormone detection systems is their portability and suitability for the construction of disposable sensors. In that aspect, the usage of screen-printed electrodes deserves particular attention due to their cost effectiveness, repeatability, and versatility, which stems from the wide variability of surfaces that can be printed on with conductive inks.^158^ Several studies describe the possibilities of using screen-printed electrodes in estrogens and progestogens determination, with their prime representatives, i.e., screen-printed carbon electrodes (SPCEs), made from carbon black, graphene, carbon nanotubes, graphite, and their derivatives. Santos et al.^164^ successfully determined EE2 at ultratrace levels by the use of (mag@MIP)-GQDs-FG-NF/SPE, i.e., a screen-printed electrode modified with functionalized graphene (FG), graphene quantum dots (GQDs), and magnetic nanoparticles coated with molecularly imprinted polymers (mag@MIP), where mag@MIP have been obtained by polymerization of methacrylic acid (monomer) with EE2 template on Fe_3_O_4_@SiO_2_-MPS particles. Similarly, a magnetic MIP system was used to modify the working electrode (screen-printed carbon electrode) for the detection of 17β-estradiol in water samples using DPV. This approach achieved a LOD of 20 nM.^165^ The screen-printed electrode rGO-AuNPs/CNT/SPE based on gold-nanoparticle-decorated reduced graphene oxide-carbon nanotubes composites has been also applied for the determination of 17β-estradiol.^166^ E2 has been also determined in environmental samples by Wong et al.^167^ who introduced a screen-printed disposable electrode modified with copper phthalocyanine, Printex 6L carbon (P6LC, carbon black), and a Nafion film (perfluorosulfonic polymer). Using the differential pulse voltammetry (DPV) technique, the sensor showed a linear concentration range between 8 × 10^–8^ and 7.3 × 10^–6^, detection limit of 5 × 10^–9^ with no measurable inferences. In turn, a reduced graphene oxide, 5-amino-2-mercaptobenzimidazole, gold nanoparticles (AuNPs) composite SPCE was used for determination of progesterone at ultratrace levels.^168^

#### Other Analytical Approaches

Chromatographic and electrochemical methods are the most popular instrumental approaches to estrogen and progestogen determination in aqueous environmental samples, but not the only ones. The spectroscopic methods seem to hold tremendous potential for environmental monitoring of sex hormones in water-based matrices because they offer high sensitivity and molecular specificity, high speed of noninvasive and nondestructive testing, and adaptability for on-site measurements. Nevertheless, their practical implementation requires addressing reproducibility and matrix effects. This group of methods includes Raman spectroscopy and its highly sensitive enhancement, i.e., surface-enhanced Raman spectroscopy (SERS), fluorescence spectroscopy, UV–vis, infrared (IR), and near-infrared (NIR) spectroscopy.

Considering the recent studies, where this group of methods has been applied, one should mention the work on MIP-coated optical progesterone sensor by Kongswang et al.^185^ The sensor was obtained by modification of glass substrate (introduction of hydroxyl group and subsequent vinylation) and, following, bonding with the MIP layer obtained taking MAA as a monomer and progesterone as a template. It can be successfully applied to aqueous progesterone solution of concentration ranging from 0.5 to 20 ng/mL with LOD = 7.4 ng/mL. Another interesting approach was presented by Qasim et al.,^186^ who fabricated hydrogel AA-based progesterone-imprinted MIP films of highly ordered 3D structures conferring them photonic properties. The MIP response was investigated with progesterone concentration in the range from 1 to 100 μg/L with an LOD of 0.5 μg/L. The developed sensor was tested in real water samples collected from wetlands near Columbia, MO, and tap water at the University of Missouri (coming from Water Treatment Plant with Columbia Utilities) with satisfactory results.

From the spectroscopic methods, SERS emerges as particularly well-suited for qualitative analysis of sex hormone traces, as shown by Wang et al.,^187^ who proposed a novel and reproducible Zn/Au–Ag/Ag sandwich-structured SERS substrate for detection of E1 and E2 in natural environmental samples. The specificity of the developed SERS-based assay stems from the very rough hierarchical structures and successful interfacial charge transfer. The SERS methodology can be effectively applied in the form of SERS-based hormone biosensors,^188,189^ discussed in more detail in the next section.

### Immunoassays, Biosensors, and Bioassays

Quantification of hormone levels in environmental water samples using analytical methods requires the use of sophisticated and expensive equipment operated by highly trained personnel. Furthermore, data interpretation requires specialized expertise and is a time-intensive process. Ongoing monitoring of hormone levels in environmental waters requires the analysis of a large number of samples. Therefore, simple, rapid methods using readily available equipment are needed for quantitative analysis. This has led to the adoption of immunoassays, a technique commonly used for physiological fluids like blood and urine.^190^

#### Immunoassays

Immunoassay techniques allow the detection of trace amounts of organic compounds (e.g., hormones) in samples and are based on the interaction of specific antibodies with an antigen (the compound whose level in the sample we want to determine). Immunoassays are usually performed on 96-well plates, which allow simultaneous analysis of multiple samples. Immunoassays can be divided by the signal emitted (RIA = radioimmunoassay; ELISA = enzyme-linked immunosorbent assay; CLIA = chemiluminescence immunoassay; ELFA = enzyme-linked fluorescence assay) or by the method of detection. Based on the detection method, there are four types of immunoassays: direct, indirect, sandwich, and competitive.^191^ In a direct immunoassay, the samples to be analyzed are placed in the wells of a plate, and then an antibody conjugated to an enzyme (or a fluorescent dye) is added. The antibody specifically binds to the antigen being labeled. After incubation, the antibody that is not bound to the antigen is removed, and a substrate is added to the well, which is converted by the enzyme conjugated to the antibody, resulting in the emission of a signal (e.g., chemiluminescent or light). The intensity of the signal is directly proportional to the amount of antigen in the sample and is measured with a plate reader. The first step in the indirect immunoassay is the same as that in the direct immunoassay, but the primary antibody is unlabeled. To determine the level of the test antigen in the sample after the unbound primary antibodies are removed, secondary antibodies conjugated to an enzyme (or fluorescent dye) that binds to the primary antibody are added to the sample. Secondary antibodies are polyclonal antibodies, which allow more than one secondary antibody to bind to the primary antibody molecule. As a result, the signal is amplified, which translates into a greater sensitivity of the method. In a sandwich immunoassay, the wells on the plate are coated with antibodies specific to the antigen under test. During incubation, the antibodies capture the test antigen from the samples. Then, unlabeled primary antibodies (specifically binding the antigen) and secondary antibodies (binding primary antibodies) conjugated to an enzyme or fluorescent dye are added sequentially. Excitation and signal reading are analogous to direct-type methods. This method is extremely precise and allows for the determination of small amounts of antigen, which makes it frequently used in diagnostics. In a competitive immunoassay, the wells on the plate are also coated with antibodies specific for the antigen being tested. In this test, an analyzed sample containing an unlabeled antigen and a known amount of labeled antigen is applied to the plate. The unlabeled antigen in the sample competes with the labeled antigen for binding sites to the antibody. Once the unbound antibody molecules have been removed, the signal emitted by the labeled antigen is measured. Since the binding of the labeled antigen to the antibody is inversely proportional to the amount of antigen in the sample, the level of the measured signal is also inversely proportional to the amount of unlabeled antigen in the sample.

A variation of immunoassays used to determine the presence of hormones in a sample is lateral flow immunoassays (LFIAs). In this test, antibodies are immobilized at a defined location on a nitrocellulose membrane embedded in a polymer chip. Once the sample is applied, capillary forces force the sample to migrate across the nitrocellulose membrane, allowing the capture of the antigen present in the sample. LFIA tests can be of sandwich or competitive type.^192^ LFIA tests are a low-cost and easy-to-perform method for determining antigen in a sample but, due to the extensive validation process, are mainly qualitative/semiquantitative.^193^

Immunoassay methods used to determine sex hormone levels in environmental waters have been validated with analytical methods and are as often used as instrumental methods such as GC or LC-MS/MS.^146^ RIA, the oldest immunological method, and ELISA, derived from RIA, are the most commonly used methods in environmental testing. RIA, due to its use of radioisotopically labeled hormones, is a highly sensitive (at the pg/mL level^194^) and quantitative technique. In addition, RIA is a competitive method; therefore, the labeled hormone is also an internal positive control for the binding of the test hormone to the antibodies used in the test. This is very important because environmental waters contain a number of substances that affect the antibody-ligand interaction. The disadvantages of RIA are the limited shelf life of the reagent, the need to work in a laboratory suitably equipped for isotope work, and the use of specialized equipment (scintillation counter). Since the radioisotope present in the test poses a risk to both humans and the environment, nowadays RIA is treated as the “gold standard” and used mainly for validation of other methods.^195^ Compared with RIA, ELISA is generally safer, cheaper, and more amenable to automation. At the same time, ELISA tests, like RIA tests, have a low detection threshold, require a small volume of sample (on the order of tens of microliters), and do not require excessive sample pretreatment, which affects the low cost of sample preparation for testing.^190^ In addition, ELISA tests: (1) are ready-to-use tests, (2) have a simple and well-defined protocol for antigen determination, (3) are easy to learn even by inexperienced individuals, (4) produce results that are easy to analyze.^190,196^ ELISA tests, however, are not without disadvantages: (1) organic and ionic compounds found in environmental waters may affect the result of the assay,^197^ (2) antibodies used in the test may interact with structurally similar compounds, including those formed by (bio)degradation of hormones,^198^ (3) highly specific antibodies that are stable in the presence of substances dissolved in environmental waters must be available,^199^ (4) the colorimetric reaction catalyzed by the enzyme conjugated to the antibody runs over time, so with prolonged incubation time the results will not reflect the level of antigen and will give a false positive result.^196^ Therefore, it is recommended that the determination of hormone levels should be performed strictly according to the accompanying protocol and on pretreatment samples.^198^ Examples of the test working range of ELISA for the determination of estrogens and progesterone in environmental water are outlined in Table 4.

**Table 4:** Examples of assay working range of ELISA for detrmination of estrogens and progesterone in environmental water

Hormone	Sample type	Sample pretreatment	LOD/LOQ/range, ng/L	determined concentration, ng/L	ref.
E1	wastewater treatment plant	filtered, SPE	50–5000	NA	(200)
E2			50–1000	2.1–202.2	
EE2			50–3000	ND–3.1	
E2	river water	filtred, DLLME	NA	5–87	(201)
EE2				10–1290	
EE2	aquaria water	filtered, SPE	6.6 × 10^3^–30 × 10^6^	600–9600	(202)
P4			7.9 × 10^3^–1 × 10^6^	75–1500	
E2	river	SPE	40 ± 20	<10	(203)
EE2	river	SPE	50 ± 10	4.1–29	(204)
P4	river, wastewater	adjusted to pH 3, filtered, SPE	ND–439	ND–3.1	(12)
E1	wastewater, river	SPE	2–4000	1–351	(205)
E2			2–4000	1–199	
EE2			2–4000	1–95	
E3			2–4000	<1–3	
P4			2–4000	0–904	

#### Biosensors

The increase in EDC levels in the environment caused the need to monitor the EDC concentrations in environmental waters. Thus, there has been a growing demand for the development of screening analytic methods that are rapid, low-cost, and feasible to perform on site.^206^ In this context, biosensor-based methods seem to be a good solution. Biosensors are characterized by high sensitivity, specificity, and miniaturization capabilities, making them ideal devices for environmental analysis.^207^

Biosensors operate based on the transduction of an electrical signal generated by redox processes occurring between the electrode (transducer) and the target analyte captured by bioreceptors immobilized on the transducer. Bioreceptors can be antibodies, nucleic acids, aptamers, molecular imprinted polymers (MIP), or whole bacterial cells.^157^ The transducer can be of electrochemical, thermal, optical, or mass-based type. Based on the operating principle of the different types of transducers, biosensors can be divided into subtypes. Optical biosensors include fiber optic, surface plasmon resonance, Raman-spectroscopy-based, and FTIR-based biosensors. Mass-based biosensors are divided into magneto- and piezoelectric biosensors. Electrochemical biosensors include potentiometric, amperometric, impedimetric, or conductometric transducers.^208^ The working principle of electrochemical transducers and the effect of the nanomaterials used on the sensitivity of transducers are described in the previous section ’Electrochemical analysis’.

The determination of EDCs in environmental waters is used primarily by immunosensors, aptasensors, and enzyme sensors.^209^ Immunosensors are devices in which the bioreceptors are polyclonal antibodies that are specific for the target analyte. Immunosensors, such as ELISA, can work with competitive, noncompetitive, direct, and indirect immunoassays.^209^ Electrochemical immunosensors achieve a sensitivity on the order of μg/L-pg/L.^207^ The disadvantages of immunosensors are the relatively high cost as a result of the cost of antibody production and purification, and the short shelf life due to the labile nature of the antibodies.^210^ In addition, antibodies are immobilized on the electrode in a random orientation that affects the sensitivity of the biosensor.^211^ In aptasensors, the biological elements that bind the target analyte are called aptamers. Aptamers are short peptides or single-stranded oligonucleotides produced by chemical synthesis. Due to their low production cost, temperature and pH stability, and insensitivity to toxicants present in complex matrices, nucleotide aptamers are mainly used in aptasensors.^206,212^ Aptamers are extremely specific, binding an antigen with high affinity (the linear response of the aptasensor for E2 is in the range pM-nM^210^). The ability of aptamers to form complex tertiary structures makes aptamers capable of recognizing subtle molecular differences within the antigen.^206^ The enzymes used in enzyme-based sensors used to determine EDCs in water are mainly tyronsinase, laccase, and xanthine oxidase.^209,213^ In the case of enzyme-based sensors, as in immunosensors, the technique of immobilizing enzymes on the electrode determines the enzymatic activity and specificity of the reaction catalyzed by the enzyme.^213^ In addition, enzyme activity is affected by the pH and temperature of the sample.^209^ The sensitivity of enzyme sensors in the determination of EDCs in environmental water samples is at the ng/mL level.^209^ Although many of the biosensors designed for EDC determination are still in the laboratory stage, work is already underway using these biosensors for environmental analysis.^207^ Examples of using biosensors for the determination of estrogens and progesterone in environmental water are outlined in Table 5.

**Table 5:** Examples of using biosensors for determination of estrogens and progesterone in environmental water

Hormone	Sample type	Bioreceptor	Detection technique	LOD/LOQ/range,	ref.
E1	river water	tyrosinase	potentiometry	0.4 ng/L	(214)
E2				0.1 ng/L	
E2	river water	aptamers	impedimetry	0.5 pM	(215)
EE2	water	aptamers	voltammetry	17 pM	(216)
P4	water	antibodies	voltammetry	15–17 pM	(217)
E2	tap water	antibodies	LSPR	11 pM	(218)
E2	wastewater	aptamers	voltammetry	0.5 fM	(219)
			impedimetry		

#### Bioassays

Analytical studies of water samples are generally restricted to quantifying the concentrations of primary compounds and, in certain instances, their known derivatives, and, thus, they do not take into account all the products of (bio)degradation and the cumulative effect of the mixture of active substances on the ecosystem.^220^ This is an important issue in hormone determination, as water samples in which the level of E2 determined by analytical methods was below the biologically relevant level (10 ng/L) showed estrogenic activity determined by the YES bioassay above the level adversely affecting aquatic organisms (>20 E2 eq ng/L).^203^ Moreover, relatively weakly active compounds (for example, E1) can be biotransformed to strongly bioactive molecules (for example, E2) in an aqueous environment.^221^ Hence, analytical testing of hormones present in water should be supplemented with bioassays, especially since a bioassay generally allows the detection of lower amounts of biologically active compounds in environmental water samples than chemical-analytical and immuno-analytical methods.^205,222^

Bioassays are experimental studies performed on living organisms (*in vivo* studies) or on cells or proteins (*in vitro* studies).^223^ Bioassays allow for the determination of the dose-dependent potency of compounds present in water on organisms.^205^ In the bioassay, the observed/measured response in an environmental water sample is comparable to the response of the experimental system to the presence of a reference compound (for estrogen testing, it is usually E2, for progestins, it is P). The estrogenic (progesterone) activity of a water sample is expressed as the equivalent concentration of the hormone under test (for estrogens: EEQ-equivalent to the concentration of E2; for progestens: PEQ-equivalent of progesterone concentration).^224^*In vivo* studies are conducted using small aquatic organisms such as *Daphnia*, algae, or bacteria.^225^*In vivo* studies are used to investigate the effects of compounds suspended in water on the behavior and condition of whole organism. *In vivo* testing also makes it possible to determine the bioavailability of substances present in a water sample.^226^*In vitro* tests are used to determine compounds in a water sample that have similar biological activities, as determined by the test. *In vivo* tests are generally conducted directly in environmental water samples. Cells used in *in vitro* tests require specific conditions, provided by the culture medium for growth. Consequently, the water sample must be specially prepared for the *in vitro* test. A less common method, called the Medium Methods, involves dissolving the components of the culture medium directly into the environmental water sample. The most common method of preparing a water sample for *in vitro* testing is the SPE technique.^227^ It is important to notice that the choice of sorbent used for SPE can affect the outcomes of bioassays. For steroid determination, samples extracted with C18 sorbent exhibited higher estrogenic activity compared to those extracted using HLB sorbent.^228^

Bioluminescent bacteria *Vibrio fischeri*, freshwater crustacean *Daphnia magna*, and aquarium fish, i.e., *Danio rerio*, are most commonly used to determine estrogen and progesterone activity in environmental water samples for *in vivo* studies.^226,229−231^ These bioassays differ in bioavailability and sensitivity to solutes in water.^231^*Vibrio fischeri* is a nonpathogenic bioluminescent Gram-negative bacterium that is used for acute toxicity testing.^232^ Tests conducted using *Vibrio fischeri* bacteria are inexpensive, rapid, and reproducible making them ideal organisms for screening.^231^ Commercially available tests based on *Vibrio fischeri* are the Microtox tests.^232^ These are inhibition tests that measure the decrease in endogenous luminescence levels of *Vibrio fischeri* bacteria induced by toxic substances present in the water sample.

*Daphnia magna* is the oldest and most widely used organism in ecotoxicological studies.^233^ The widespread use of *Daphnia magna*-based tests is due to the sensitivity and ease of culturing these organisms. Additionally, *Daphnia magna* is relatively inexpensive.^226^ Other advantages of *Daphnia magna* include size (a parameter linked to the ability to accumulate toxins and the ability to culture several *Daphnia magna* on a single culture plate), a short life cycle, ease of observing various parameters without the need for invasive methods due to the transparency of the body and the use of immobilized organisms, parthenogenetic reproduction, and high fecundity that allows for a rapid increase in the number of individuals in culture.^233^ The parameters most often used as indicators of toxicity in tests based on *Daphnia magna* are immobilization, lethality, locomotorisity, and reproductivity.^229^

*Danio rerio* is a highly characterized model organism recommended by the International Organization for Standardization for use in determining the toxicity of compounds present in environmental waters.^234^ Its advantages include: (1) high reproduction rate, (2) rapid growth, (3) short life cycle, (4) undemanding diet that makes breeding economically viable, (5) ease of introducing genetic changes (tests often use transgenic individuals with a reporter gene inserted into the genome), and (6) tests are conducted on both male and female individuals.^234,235^ However, tests based on *Danio rerio* have relatively low sensitivity and high variability, due in part to the fact that sex influences an individual’s response to contaminants.^235^ Ecotoxicological tests are conducted mainly on individuals in the early stages of development (embryonic stage, embryolarval) because juveniles are transparent (expression of reporter genes is easily observed by simple methods, such as fluorescence microscopy) and small (tests can be conducted on microplates using small volumes of environmental water samples). In addition, fish embryos are less sensitive to pain and stress.^236^

*In vitro* bioassays are called bioanalytical tools because they are capable of determining trace amounts of compounds in a complex mixture that exhibit specific biological activity.^205,222^ Compared to *in vivo* testing, assay, an *in vitro* assay is more uncomplicated, cost-efficient, and time-saving to conduct.^226^ Poor standardization of the method and the possibility that toxic compounds in the mixture interfere with the measurement are cited as disadvantages of *in vitro* testing.^222^*In vitro* studies of the steroid activity of compounds are based on the canonical (genomic) pathway of action of steroid hormones such as estrogen or progesterone.^224^ Shortly, in this pathway, compounds with steroid hormone activity bind to the appropriate nuclear receptor found in the membrane, cytoplasm, or cell nucleus.^237^ Binding of the steroid hormone activates the receptor, allowing for dimerization of the receptor. The resulting homodimer is a transcription factor that is transported to the nucleus. In the nucleus, the homodimer binds to specific sequences (EREs - estrogen response elements; PREs - progesterone response elements) or to other transcription factors that activate the expression of genes whose expression is regulated by steroid hormones.^238^ Steroid hormones can also act through nongenomic pathways, that is, binding to membrane G protein-coupled hormone receptors. Activation of these receptors induces an increase in the concentration of NO or Ca^2+^ in the cell cytoplasm.^237^ Although activation of the nongenomic steroid hormone pathway does not activate the *in vitro* response of compounds with steroid hormone activity, it can influence the outcome by involving compounds with steroid hormone activity in the nongenomic *in vitro* assay-specific response.

*In vitro* bioassays used to determine sex hormone activity in environmental water samples are based mainly on human cells or yeast cells.^224^ These tests measure a change in a cell’s physiological process or use a reporter gene whose expression is under the control of the sex hormone. An example of an *in vitro* bioassay that measures a change in the physiological process of the cell is the quantitative E-SCREEN assay. The E-SCREEN assay takes advantage of the fact that the proliferation of human breast cancer MCF-7 cells is regulated by estrogens. The E-SCREEN assay compares the number of MCF-7 cells growing in estrogen-free medium with the number of MCF-7 cells growing in the presence of different concentrations of E2 (positive control) or different dilutions of environmental water samples.^239^ Another MCF-7 cell-based test is the MELN assay. MELN is a reporter cell line derived from MCF-7 cells stably transfected with a reporter gene (gene encoding luciferase) expressed under the control of estrogen receptor α. Activation of endogenous estrogen receptors induces luciferase expression. Upon the addition of a substrate, luciferase catalyzes a reaction in which readily measurable luminescence is produced.^240^ The amount of luminescence produced is proportional to the amount of hormone-specific receptor activation. It should be noted that methods based on cells with stable expression of reporter genes are easy to implement in the laboratory and allow specific, sensitive, and reproducible determination of the test substance.^241^ Therefore, tests based on stably transfected cells are often used to test environmental samples. A popular test for determining the activity of steroid hormones in water is CALUX (chemically activated luciferase expression) test. The CALUX system is human cell lines (usually derived from the human osteosarcoma U2OS cell line or the human breast cancer T47D cell line) stably transfected with a gene encoding luciferase containing three receptor binding sites for the sex hormone under test and a minimal promoter.^227^ The CALUX bioassay uses endogenous hormone receptors (e.g., the ER-CALUX assay derived from the T47D line measuring estrogen activity^148^) or cells are cotransfected with a gene encoding a human steroid hormone receptor (e.g., the PR-CALUX assay derived from the U2OS line measuring progesterin activity^242^). In the CALUX assay, as in the MELN assay, the activity of sex hormones is proportional to the level of luminescence produced by luciferase, the expression of which is induced by the sex hormone under test. The activity of sex hormones present in water can also be determined by measuring the fluorescence. GeneBlazer bioassay works on this principle. GeneBlazer technology is based on human kidney cells HEK293 stably transfected with a reporter gene encoding β-lactamase (bla gene) and cotransfected with a gene encoding a receptor for the test steroid hormone. Expression of the bla gene is controlled by the active receptor for the test hormone.^240^ Detection of hormone activity in the GeneBlazer is a FRET (Fluorescence Resonance Energy Transfer)-based assay. Briefly, after incubation with the test sample, a substrate, which easily diffuses through the cell membrane and consists of two fluorophores (coumarin and fluorescein), is added to the cells. These fluorophores are linked together to form a FRET pair. In cells without β-lactamase, excitation of coumarin causes FRET to the fluorescein and emission of green light. In the presence of β-lactamase, the substrate is cleaved, causing the fluorophores to move away from each other, making FRET impossible. As a result, coumarin excitation induces blue fluorescence emission. The expression level of the bla gene (proportional to the level of the substance with the activity of the measured hormone) is quantified by measuring the ratio of blue to green fluorescence.

The determination of sex hormone activity by a human cell-based bioassay requires cell culture experience. In addition, cell cultures are sensitive to environmental conditions and require a complex culture medium.^243^ The average life cycle of a cultured human cell is several hours, so obtaining the pool of cells necessary for the test is time-consuming. Moreover, sex hormones are natural molecules that regulate human cell processes and are metabolized in these cells, which means that physiological cellular processes affect the result obtained in human cell-based bioassays.^226,244^ These limitations are not possessed by yeast cells, which are easy to culture, grow in a simple, hormone-free medium, have a short cell cycle (tens of minutes), and do not have endogenous sex hormone receptors.^243^ Due to their low cost and low apparatus requirements, yeast cell-based bioassays are widely used to monitor the activity of sex hormones in environmental waters.^227^ The most widely used method, the yeast screen assay, is based on a recombinant yeast strain *Saccharomyces cerevisiae* with an introduced constitutively expressed gene that encodes the human estrogen receptor (YES assay) or the progesterone receptor (YPS assay) and a gene that encodes β-galactosidase. The expression of β-galactosidase is induced by the active estrogen/progesterone receptor. Synthesized in the presence of the appropriate sex hormone, β-galactosidase transforms the substrate into a colored product whose concentration is proportional to the level of the hormone.^228^ When choosing a cellular system to study the activity of sex hormones, it is important to take into account that cells differ in their sensitivity to a particular hormone. Studies conducted by Kunz^224^ with 5 different bioassays (GeneBlazer-ERα; YES; ERα-CALUX, MELN, T47D-KBluc) showed that the response of human cell lines to estrogens or compounds exhibiting estrogenic activity varies and depends on the type of substance. In addition, the reproducibility of the results depended on the assay used and was best for the ERα-CALUX and YES assay.^224^ However, note that yeast cells are less sensitive to sex hormones. Differences in metabolism and limited cell wall permeability to steroid hormones have made yeast cells not able to be recommended for hormone activity testing.^227^ However, research is underway to obtain recombinant yeast lines with improved permeability and sensitivity to steroid hormones.^245^ Examples of using *in vitro* bioassaying for determination of estrogens and progesterone in environmental water are described in Table 6.

**Table 6:** Examples of using *in vitro* bioassaying for determination of estrogens and progesterone in environmental water

Hormone	Sample type	assay	LOD/LOQ/range,	ref.
P4	wastewater	PR-CALUX	<0.17 ng/L	(246)
E1, E2, EE2, αE2	wastawter	ERαCALUX	ND	(247)
E2	river	ER-GeneBlazer	ND-6 ng/g	(248)
P4	river	PR-GeneBlazer	ND-0.04 ng/g	
E2	river	MELN	0.002	(248)
E2	reused water	YES	12 ng/L	(249)

## Discussion

The ubiquitous pollution of environmental waters by steroid hormones, including estrogens and progestagens, can be considered a sign of contemporary times. Moreover, the risk associated with environmental contamination by sex hormones is still difficult to assess due to their low concentration and the lack of or only scarce data for single compounds and, especially, for their mixtures, transformation products, and metabolites. The question of the endocrine burden of the EDCs from environmental waters is also insufficiently studied. This problem stimulates dedicated multidisciplinary research and the development of procedures for the management and control of pollutants, which should report not only the levels of the most potent endocrine disruptors but, complementarily, also net biological effects exerted by all EDCs present in the sample (together with the unidentified ones). These tasks require tailored analytical approaches to sample collection, transport, preparation, and finally hormone quantification and/or estimation of its biological potency. In turn, a reasonable selection of the analytical strategy should take into account the expected sensitivity and selectivity of the determination, its speed and throughput, the trace levels of the analytes, their stability, and possible matrix effects.

In this review, we outlined the dominant established methods used in the estrogens and progestogens analytics, together with their recent significant improvements or synergistic approaches, that can be applied in monitoring of environmental waters, both at the level of the quantitative evaluation of considered sex hormones and their biopotency assessment. Particular attention was paid to the challenges related to sample preparation. It is a critical stage of the analysis that can affect the accuracy, precision, reliability, and repeatability of the results. The review emphasizes the importance of proper sampling, transportation, storage, preprocessing (including, e.g., filtration), and extraction of analytes, which can prevent underestimations in endocrine activity and inaccuracies in the determination of hormone concentration due to loss, degradation, or interference of analytes. The preprocessing of real water samples involves numerous challenges stemming from the specific properties of sex hormones and the complexity of the water matrix. The literature highlights the following key issues. First, steroid hormones occur in the aquatic environment in very low concentrations, often at the ng/L or even the pg/L level (Table 1). Second, there is the question of matrix complexity. Water samples, particularly wastewater, contain many interfering substances that can hinder the isolation and detection of steroid hormones. Third, there is a question of the stability of estrogens and progestagens. Steroid hormones are prone to degradation under the influence of light, pH, temperature, and microorganisms. Therefore, maintaining optimal conditions for sample storage and preprocessing is one prerequisite for reliable analysis.

In Table 7, we show the list of the most commonly used sample preconcentration techniques, together with bullet points on their advantages and limitations. Among them, SPE is preferred for most routine analyses because it offers a good balance of selectivity, efficiency, and cost, as well as its compatibility with most analytical methods for the determination of analytes.^117,118^ This approach is versatile because of the broad range of sorbent and solvent types and the possibility of flexible optimization of extraction parameters, which, after proper adjustment, can fulfill the actual experimental needs in terms of the required sensitivity, simultaneous analyses of multiple analytes, etc. The still popular classical hydrophobic sorbents (C_8_, C_18_)^24,150,152^ and the hydrophilic–lipophilic balance sorbents (HLB), being most commonly used for natural and synthetic hormones extraction in aqueous environmental samples,^21,27,86,87^ can suffer from matrix effects, which affect the analyte signal compared to the real concentration.^85^ To minimize this drawback, novel combined and/or analyte-tailored SPE solutions have been developed. In this respect, using multiple-sorbent SPE can efficiently improve the performance and reliability of hormone quantification in real-life samples.^23^ Among the sex-hormone-oriented customized SPE solutions, which turned out to be successful in sex hormone analytics, one should mention above all functionalized metal–organic frameworks,^89,90^ functionalized mesoporous silicas,^101,104,105^ molecularly imprinted polymers^98,99^ including the MIP-based cartridges available on market (Affinimip SPE Estrogens from Affinisep, Val-de-Reuil, France)^100−103^ and magnetic nanoparticle-based materials, which frequently are also MIP-composites.^91−96^ Considering the common drawbacks of these technologies, one should mention the possible incomplete template removal for some MIP-based solutions due to their high binding affinity and slow-release kinetics, which may, eventually, lead to false positives or at least a time-consuming analytic process.^169^ Historically, some MIP sorbents have exhibited reduced permeability, necessitating the use of relatively large amounts of sorbent. Such problems commonly occur in the case of MIPs obtained by bulk polymerization. These drawbacks stem from the irregularity of the polymer particles’ sizes and the loss of binding sites or their hard-to-reach location due to the grinding and sieving of the polymer blocks before their SPE application.^120^ MIP particles obtained by suspension or precipitation polymerization yield uniformly shaped polymer particles, but these still do not meet restrictive expectations in sample separation from complex environmental matrices. Finally, surface molecular imprinting has offered a possible solution to avoid these problems, because it gave the possibility to produce a tailored core–shell system allowing one to reach more effective recognition sites and faster mass transfer.^108^ These core–shell structures have built up the broadest and most robust family of MIP-based SPE sorbents due to the plethora of available 1D, 2D, and 3D nanoarchitectures ensuring high availability of affinity sites for hormone binding,^157^ and when coupled with, e.g., a magnetic nanoparticle core one can reach both high surface contact, full interaction between the analytes and the sorbent particles, as well as easy and rapid transfer and isolation of sorbed analyte from the solution using an external magnetic field.^119,120^ The high binding affinity of the MIP-sorbents, similar to other functionalized sorbents or immunosorbents, is critical for reaching picomolar–nanomolar limits of detection of estrogens and progestagens in environmental waters. This feature is crucial for both analyte extraction and, e.g., electrochemical sensing devices, and it is often correlated with the high specificity and sensitivity of the analyte determination. Nevertheless, from the perspective of longitudinal, multicycle, and stable monitoring, full reversibility of the analyte binding is also important. Thus, among formidable engineering challenges in the field, one of them is ensuring fast and controllable on–off kinetics of analyte binding to the sorbent. In that aim, stimuli-responsive materials can be utilized, which can change their physicochemical properties in response to external factors such as temperature, light, magnetic or electric fields, pH, biomolecules, ions, solvents, allowing for the effective release of the target molecule from the sorbent matrix.^250−253^ Although the stimuli-responsive materials have been proven to be a possible solution to tackle the trade-off between binding affinity and on/off kinetics for capturing biomolecules like dopamine^253^ or albumin,^250^ there is still an open niche for their sex-hormone applications.

**Table 7:** Most common preconcentration methods that can be applied before hormone determination in aqueous environmental samples, together with indication of their typical sorbents, advantages, limitations, and applicability for other matrices

Abbr.	Method	Type of sorbent (examples)	Advantages/Limitations	Applications
SPE	solid phase extraction	hydrophobic sorbent (C8, C18), hydrophilic–lipophilic balance (HLB), MIP etc.	advantages: • compatible with most analytical techniques (e.g., UHPLC, LC, GC) • simplicity, selectivity, versatility, • possibility of process automation • possibility of adjusting sorbents • effective removal of interferences • effective enrichment of the analyte with hormones; limitations: • use of organic solvent • time-consuming • possible analyte losses	environmental samples, body fluids, food
SPME	solid phase microextraction	thin fiber covered with sorbent	advantages: • miniaturized, solvent-free SPE variant • faster and more eco-friendly than regular SPE • smaller apparatus than used for SPE, allowing portability; limitations: • low mechanical durability of fibers	environmental samples, food, soil, air, body fluids
MSPE	magnetic SPE	magnetic molecularly imprinted composite nanoparticles	advantages: • short extraction time, • less adsorbents required in comparison with conventional SPE; limitations: • selectivity problems for some adsorbents	environmental samples, body fluids, food, drinks
LLE	liquid–liquid extraction	organic solvents	advantages: • simple implementation of equipment and processes; limitations: • use of large amounts of organic solvents	environmental samples, liquid food samples
LPME	liquid-phase microextraction	organic solvents, DESs, ILs	advantages: • small volumes of organic solvents • eco-friendly • simple and inexpensive; limitations: • limited sample volume—only for low sample volumes	environmental water and solid samples, biological samples, food
IA-SPME	immuno-affinity extraction	immobilized antibodies	advantages: • low solvent usage • high selectivity • eco-friendly; limitations: • need for specific antibody production	environmental samples, body fluids, food, drugs
QuECh- ERS	quick, easy, cheap, effective, rugged, and safe	acetonitrile, methanol, ethyl acetate	advantages: • quick and simple metod • suitable for extracting a broad range of analytes • high efficiency • eco-friendly; limitations: • enrichment of analytes • loss of some extractable substances	environmental samples, biological samples, food, drugs

Among current trends in analytical chemistry, greater focus is being placed on green methods to minimize the environmental impact. The sex hormone analytics in environmental water samples is no exception in this respect.^83^ While some methods of estrogen and progestogen determination effectively implement sustainable principles by reducing or removing the sample preparation (like, e.g., the electrochemical approaches), frequently it remains a necessary step for complex matrices (e.g., water reach in suspended solids and organic matter), when dealing with (ultra) traces, and/or when the determination method requires precedent sample derivatization (like GC-MS). Among the sample preparation methods being in line with the principles of green chemistry, one can indicate, e.g., SPME,^88^ IA-SPME,^61^ QuEChERS,^62,140^ QuEChERS with SS-LPME,^142^ QuEChERS with SPE,^143^ thin-film microextraction,^30,31^ and membrane-assisted solvent extraction.^254^ In addition, microextraction methods using ionic liquids^137^ or deep eutectic solvents^138,139^ are noteworthy. These methods allow for significant reductions in reagents and energy consumption and consequently a decrease in hazardous waste, sometimes for a price of reduced sensitivity compared to the more traditional extraction methods, including SPE or LLE.

Through the years, various analytical techniques have been utilized in the determination of steroid hormones in real aqueous samples, which significantly differ with the sensitivity and specificity, liability to matrix effects, equipment and training requirements, cost-effectiveness, ease of implementation, scalability, etc. Frequently, the chromatographic analysis coupled with mass spectrometry remains a gold standard for the qualitative and quantitative estrogens and progestogens analysis in complex environmental matrices^82^ despite its relatively high instrumentation costs, labor complexity, and time-consumption. Possible solutions to the listed problems are offered by the online SPE-LC-MS/MS systems, automated extraction platforms, and modern analytical instruments, which enable faster, more efficient, and economical hormone analysis. The chromatographic approaches exceed other methods in the number of compounds that can be simultaneously analyzed with maintaining (ultra)trace determination levels and good separation capabilities, even in case of structural analogs, as shown, e.g., by Zhao et al. determining 24 steroid hormones,^91^ Grobin et al. monitoring 25 EDCs,^59^ Zhang and Fent analyzing 33 steroids,^21^ Shen et al. considering over 60 natural and synthetic progestins.^87,151^ On the contrary, the abilities for simultaneous detection of estrogens and/or progestagens by other approaches are restricted to several compounds for electrochemical methods,^162^ SERS-based ones,^187^ or aptasensors.^255,256^ Thus, these methodological approaches rather serve for a group-targeted testing^174,255,256^ with a prominent representative of oligonucleotide editing technology upgrading the aptamer-based testing, which was successfully utilized for one-step detection of coexisting estrogens (E1, E2, E3, and EE2) in real aqueous samples.^257^ On the other hand, the nonsuitability of electrochemical nanosensors for the simultaneous quantification of individual concentrations of steroid hormone derivatives, and its overall sensitivity to interferences in complex real water matrices, can be considered as one of the most significant limitations of this group of methods. Overcoming that problem may be a prospective subject for further studies.

Considering further electrochemical biosensors, they have similar sensitivity to chromatographic – mass spectrometry techniques without the high cost and training requirements. Moreover, they can be miniaturized and utilized as portable sensors for fast, sensitive, easy, on-site hormone monitoring in environmental waters just after sample collection, as postulated by Li et al.^258^ Microfluidic innovations and nanotechnology advances foster the design of (nano)sensors, which are widely developed for diagnostic applications, e.g., for pregnancy monitoring.^259^ Hopefully, they can be successfully adapted for environmental tracing, where fast and easy procedures for determination of hormones become necessary also in the context of legislation (e.g., implementation of the new water EU directive and presence of the E1, E2, and EE2 compounds on the list of “Environmental quality standards for priority substances in surface waters”). Considering the question of rapid and reliable testing of environmental samples, another challenge is the identification and quantification of hormone transformation products formed in the environment as well as during wastewater treatment processes, in physicochemical, biological, and advanced oxidation processes. Most of the chemical analyses are restricted to several estrogens (E1, E2, E3, and EE2) and progesterone, whereas their derivatives are not considered. One of the possible approaches to address the problem is to carry out parallel water testing by bioassays. They describe the effects of total sex hormone load (exerted by known and unknown EDCs), giving complementary information for quantification of key estrogens and progestagens by analytical methods. What is also worth mentioning, biological assays, similarly to nanosensors, bring the possibility of rapid and sensitive testing (even under detection limits of analytical methods) that can be utilized for frequent and large-scale screening.

## Conclusions

This work outlines the main aspects of estrogen and progestogen analytics and bioscreening, which matches the contemporary challenges of environmental water monitoring. Undoubtedly, the presence of hormones in aquatic environments poses a serious threat to human health and the balance of ecosystems. Their monitoring is therefore crucial, and the development of sensitive and selective analytical methods should be a priority. Although analytical techniques such as HPLC combined with MS/MS offer high efficiency, researchers are still faced with challenges in finding methods that allow minimization of the influence of the matrix, increasing the sensitivity and selectivity, and standardization of procedures. Systematic and reliable assessment of the biological activity of water bodies contaminated with hormones and their versatile derivatives should also be considered as a key focus. The aforementioned determination of hormones in environmental samples and evaluation of their endocrine disrupting potency require the collaboration of experts from various fields, including analytical chemistry, biology, toxicology, and environmental engineering. An interdisciplinary approach is essential to developing comprehensive strategies for monitoring and reducing environmental hormone pollution. From the scientific perspective, the choice of appropriate methodology for hormone quantification and evaluation of their biological burden is crucial for the further research on the accumulation and transformations of sex hormones in the environment and long-term risks of exogenous EDCs intake for humans and fauna. As long as the mitigation of micropollutants remains suboptimal, academic interest in these subjects is expected to endure.

## Abbreviations Used

17OHP: 17-hydroxyprogesterone
3DOC*: triplet dissolved organic carbon
3DOM*: triplet state dissolved organic mater
A: androstenedione
AA EQS: annual average value environmental
AALLME: air-assisted liquid–liquid microextraction
APCI: atmospheric pressure chemical ionization
ATPS: aqueous two-phase system
AuNPs: gold nanoparticles
BAμE: bar adsorptive microextraction
BPA: bisphenol A
BSTFA: N,O-bis(trimethylsilyl)-trifluoroacetamide
CALUX: chemically activated luciferase gene expression
CB: carbon black
CCSHLLE: current salting-out homogeneous liquid–liquid extraction
CLIA: chemiluminescence immunoassay
CNTs: carbon nanotubes
CSA: *l*-menthol and (1*S*)-(+)-camphor-10-sulfonic acid
CV: cyclic voltammetry
D5: decamethylcyclopentasiloxane
DAD: diode array detector
DCM: dichloromethane
DES: deep eutectic solvents
DES*: diethylstilbestrol
DES-LPME: deep eutectic solvent based liquid phase microextraction
DLLME: dispersive liquid–liquid microextraction
DNS-Cl: dansyl chloride
DPAdSV: differential-pulse adsorptive stripping voltammetric
DPV: differential pulse voltammetry
DRO: drospirenone
DVA: 2,5-divinylterephthalaldehyde
E1: estrone
E2: 17β-estradiol
E3: estriol
EBM: effect-based method
ECL: electrochemical luminescence
EDC: endocrine disrupting chemical
EE2: 17α-ethinylestradiol
EEQ: equivalent to the concentration
EGDMA: ethylene glycol dimethacrylate
EI: electron ionization
EIS: electrochemical impedance spectroscopy
ELFA: enzyme-linked fluorescence assay
ELISA: enzyme-linked immunosorbent assay
EQ: equivalent of progesterone concentration
EQS: environmental quality standard
EREs: estrogen response elements
ESI: electrospray ionization
FD: fluorescence detection
FG: functionalized graphene
GCE: glassy carbon electrode
GC–MS: gas chromatography–mass spectrometry
GC–MS/MS: gas chromatography-tandem mass spectrometry
GNP: gold nanoparticle
GO: graphene oxide
GQDs: graphene quantum dots
HEK293: human kidney cells
HLB: hydrophilic–lipophilic balance sorbents
HPLC: high performance liquid chromatography
HPLC-UV: high-performance liquid chromatography coupled with ultraviolet detection
HRGC-(NCI)-MS: high-resolution gas chromatography with negative chemical ionization mass spectrometric detection
IAC: immunoaffinity chromatography
ILs: ionic liquids
IR: infrared spectroscopy
K: partition coefficients
LC-HRMS: liquid chromatography-high resolution mass spectrometry
LC–MS: liquid chromatography–mass spectrometry
LC-MS/MS: liquid chromatography-tandem mass spectrometry
LC-Q-TOFMS: liquid chromatography-quadrupole-time-of-flight mass spectrometry
LDH: layered double hydroxide
LFIA: lateral flow immunoassays
LLE: liquid–liquid extraction
LLME: liquid–liquid microextraction
LNG: levonorgestrel
LOD: limit of detection
LOQ: limit of quantification
LSPR: localized surface plasmon resonance
MAA: methacrylic acid
mAbE: monoclonal antibody
MAC EQS: maximum allowable concentration environmental quality standard
MCF-7: the human breast cancer cell line
MEKC: micellar electrokinetic chromatography
MGA: megestrol acetate
MIL: magnetic ionic liquids
MIP: molecularly imprinted polymer
MISPE: molecularly imprinted solid-phase extraction
MMIP: magnetic molecularly imprinted polymers
MMM: mixed-matrix membrane
MPs: magnetic particles
MRM: multiple reaction monitoring
MSPE: magnetic solid phase extraction
MSTFA: *N*-methyl-*N*-(trimethylsilyl)trifluoroacetamide
MTBE: methyl *tert*-butyl ether
MWCNTs: multiwalled carbon nanotubes
ND: not detected
NG: norgestrel
NIR: near-infrared spectroscopy
NIST: National Institute of Standard and Technology
NTR: norethisterone
P: progesterone
PANI: polyaniline
PIL: protic ionic liquid
PPy: polypyrrole
PREs: progesterone response elements
PSA: Primary and Secondary Amines
QTOF-MS: quadrupole time-of-flight mass spectrometry
QuEChERS: quick, easy, cheap, effective, rugged, and safe
RAFT: reversible addition–fragmentation chain transfer
RET: fluorescence resonance energy transfer-based assay
RGO: reduced graphene oxide
RIA: radioimmunoassays
ROS: reactive oxygen species
RSD: relative standard deviation
SADF-LPME: spray-assisted liquid-phase microextraction based on droplet formation
SBF: simulated body fluid
SBSE: stir-bar sorptive extraction
SERS: surface-enhanced Raman spectroscopy
SIA-LOV: sequential injection analysis-lab on valve system
SIM: selected ion monitoring
SPCE: screen-printed carbon electrode
SPE: solid phase extraction
SPME: solid-phase microextraction
SPWE: screen-printed working electrode
SS-LPME: switchable solvent-based liquid phase microextraction
SWV: square wave voltammetry
T47D: the human breast cancer cell line
TF-ME: thin film microextraction
TLL: tie-line length
TMCS: trimethylchlorosilane
TRIM: trimethylolpropane trimethacrylate
TST: testosterone
U2OS: the human osteosarcoma cell line
UA-DMSPE: ultrasound-assisted dispersive magnetic solid phase extraction
UAE: ultrasound-assisted extraction
UFLC-MS/MS: ultrafast liquid chromatography-tandem mass spectrometry
UHPLC: ultrahigh pressure liquid chromatography
UPLC: ultrahigh performance liquid chromatography
WWTP: wastewater treatment plant
αE2: 17α-estradiol
γ-MAPS: 3-(trimethoxysilyl)propyl methacrylate
μLD: microliquid desorption
μSPE: microsolid phase extraction
